# Cationic and Nonionic Surfactant Micelles in a Halogen-Free Carboxylic Acid-Based Deep Eutectic Solvent

**DOI:** 10.1021/acs.langmuir.4c05370

**Published:** 2025-05-13

**Authors:** Elly K. Bathke, Sylvain Prévost, Fátima Herranz-Trillo, Subramee Sarkar, Laura Deeming, Ronak Kakadiya, Maggie Kroon, Daniel T. Bowron, Karen J. Edler

**Affiliations:** † Centre for Analysis and Synthesis, Department of Chemistry, 5193 Lund University, Naturvetarvägen 22, Lund 223 62, Sweden; ‡ Institut Laue-Langevin, 71 avenue des Martyrs, Grenoble 38042, France; § MAX IV Laboratory, Fotongatan 2, Lund 224 84, Sweden; ∥ Science and Technology Facilities Council, ISIS Neutron and Muon Source, Rutherford Appleton Laboratory, OX11 0QX Didcot, Oxfordshire, U.K.

## Abstract

In recent years, it has been shown that deep eutectic solvents (DES) and similar mixtures solvate and allow for self-assembly of surfactants, serving as potential “green” alternatives as solvents for, for example, templating nanomaterials or drug delivery applications. Which surfactants are soluble and how they self-assemble depends strongly on the mixture components and their molar ratio. Here, we present the surfactant behavior in halogen-free citric acid: glycerol-based systems and show how a change in the molar ratio can affect the micellization of cationic surfactants. We also study micellization of nonionic ethylene oxide surfactants, which are insoluble in the most common hydrophilic choline chloride-based DES, such as choline chloride: urea and choline chloride: glycerol, in the absence of water. We find that the cationic C_12_TANO_3_ and C_16_TANO_3_ form spherical micelles with significantly higher intermicellar interactions than in comparable choline chloride-based DES, indicating that less charge screening due to the solvent components takes place. The nonionic Brij L23 (main component C_12_EO_23_) is also found to form spherical micelles in 1:2 citric acid: glycerol, while the nonionic Brij L4 (main component C_12_EO_4_) forms less clearly structured phases at similar concentrations.

## Graphical abstract

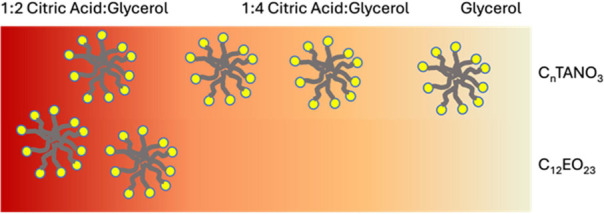

## Introduction

Solvents are an essential part of modern chemical processes and industrial production, and with the rise of safety and environmental considerations, the search for new solvent systems that enable a different approach to traditional solvents is always a goal in research. Deep Eutectic Solvents are a class of novel solvents that can be produced by mixing two or more components, at molar ratios at, or close to, the eutectic point. Strong interactions between the components result in melting points in these mixtures lower than those predicted for an ideal mixture. There is particularly high interest in those mixtures that form liquids around room temperature, as many can be useful due to their wide range of tunable properties. They can be formed from many different types of molecules; sugars, metal salts, amino acids and various other organic molecules and salts.^1,2^ Using parameters such as different components, molar ratios, and the use of additives, the solvent properties of these DES and similar low melting temperature mixtures have been shown to be adjustable to various applications from extraction to material synthesis.^1,3,4^ This gives them the potential for use as “designer solvents”, similar to ionic liquids, where the solvent properties can be optimized to the application. Many of the components are nontoxic, biodegradable, can be sustainably sourced, and are already produced on an industrial scale,^4–6^ and this makes them popular choices as safer and more environmentally friendly, modern solvents.

Surface active agents, or surfactants, are amphiphilic molecules usually consisting of a hydrophobic, hydrocarbon-based “tail” region and a hydrophilic “head” group. They can be used directly by the consumer, in soaps and other cleaning products, and also have various other uses, e.g., in emulsions in skincare products, in and with biological systems, for chemical compartmentalization, and structural templating of particles.^7–9^ Surfactants are often categorized based on their charge, such as cationic, anionic, nonionic and zwitterionic, as well as their tail length, headgroup type, and potential counterion, e.g., the common cationic surfactant hexadecyltrimethylammonium bromide (C_16_TAB) has a linear tail comprising 16 carbons, a trimethylammonium headgroup, and a bromide counterion. In solution, they have the ability to self-assemble into structures of different shapes and sizes, such as micelles, lamellae, and liquid crystal phases, depending on the amphiphile and the solvent properties.^10^ Their use is very well established, and the emergence of new solvent types, such as DES, has led to research into new possibilities. In these new solvent types, studies into both the self-assembled structures and the factors influencing them,^11,12^ as well as applications such as electrodeposition,^13^ medical applications,^12,14^ and templating nanomaterials^15,16^ have been performed.

In this paper we present the adjustable solubilization and self-assembly of the nonionic surfactants Brij L23 (main component C_12_EO_23_) and Brij L4 (main component C_12_EO_4_), and the cationic surfactants C_12_TAB and C_12_TANO_3_, as well as C_16_TAB and C_16_TANO_3_ in halogen-free solvents based on citric acid (CA) and glycerol (Gly), through fluorescence and small angle neutron and X-ray scattering. We show how the molar ratios of the components can influence their solubility and self-assembly. Most studies on self-assembly in similar systems have been performed in choline chloride-based DES, but these systems can also be limited by the presence of halogen ions, which tend to have strong interactions with other components in the system and have been shown to, for example, poison catalytic processes. Choline chloride (ChCl) also shows significant ester formation in mixtures with carboxylic acids, leading to the formation of water, which has to be considered for their use.^17^ Common ethylene oxide-based nonionic surfactants show low or no solubility in most of the frequently studied ChCl-based mixtures, although these nonionic surfactants have been found to be soluble in DES based on cerium nitrate and urea, hydrated DES, and in hydrophobic DES.^18–21^ We find that the nonionic surfactants Brij L23 (also known as “Brij 35”, main component C_12_EO_23_) and Brij L4 (mainly C_12_EO_4_) are soluble and self-assemble in CA:Gly at the molar ratio of 1:2, but not in 1:4 or neat glycerol. We also show how using different solvents, 1:2 and 1:4 CA:Gly, as well as neat glycerol, affects the self-assembly of C_12_TANO_3_ and C_16_TANO_3_.

## Experimental Section

### Materials

Hydrogenated citric acid (>99%), glycerol (>99%), Brij L4, Brij L23, sodium dodecyl sulfate (>99%), C_12_TAB (dodecyltrimethylammonium bromide) (>98%) and C_16_TAB (cetyltrimethylammonium bromide) (>98%), silver nitrate (>99%), sodium hydroxide (>98%), ethanol (>98%), pyrene (>99%), and concentrated nitric acid solution (>65%) were purchased from Sigma-Aldrich and used without further purification. Sodium lauroyl sarcosinate (>97%) was purchased from Fisher Scientific and used without further purification. Amberlite IRN-78 (OH-form) was purchased from Acros Organics. C_12_TACl (dodecyltrimethylammonium chloride) (>98%) and C_16_TACl (cetyltrimethylammonium chloride) (>98%) were also purchased from Sigma-Aldrich and used solely for ion-exchange to C_12_TANO_3_ and C_16_TANO_3_, respectively. Details can be found in the Supporting Information. Fully deuterated d8-glycerol (>99%, 99.6 atom% D) and D_2_O (>99%, 99.6 atom% D) were purchased from ckisotopes and used as provided. Deuterated d4-citric acid (>99%, 95 atom% D) was generated using lyophilization of the hydrogenated compound, which was recrystallized three times in D_2_O. ^1^H NMR on a 400 MHz Bruker Neo instrument was used to determine the degree of deuteration of the d4-citric acid. Fully deuterated C_12_TAB and C_16_TAB (>98%) were provided by the ISIS Deuteration Facility and ion-exchanged in the same way as the hydrogenated form.

### Sample Preparation

The solvents were prepared by mixing the components at 60 °C for up to 24 h until a homogeneous liquid was formed. Cationic surfactant solutions were produced by mixing the appropriate amount of DES and surfactant at 50 °C for at least 12 h, and nonionic samples were prepared through mixing at room temperature for at least 24 h.

The sample preparation method for the samples used in the fluorescence measurements can be found in the Supporting Information.

Four contrasts of the 1:2 citric acid: glycerol solvent were produced for the small-angle neutron scattering measurements: H:H, H:D, D:H, D:D. All the scattering length densities (SLDs) and contrasts with surfactants used are listed in the Supporting Information (Table S2a,b).

### Methods

Small-angle neutron scattering (SANS) experiments were conducted at the Institut Laue-Langevin (ILL), Grenoble, France, on the D11 beamline, using multiple sample-to-detector distances (1.7, 5.5, 16.5, 30 m), covering a Q-range of 0.0006–0.53 Å.^22^ Samples were measured in 1 mm path length rectangular quartz cuvettes (Hellma GmbH) inside a temperature-regulated sample changer at 25 °C, and 50 °C for the C_16_TANO_3_ samples due to their increased Krafft temperature. Additionally, measurements were taken of the empty beam, an empty cell, and the neat solvent and used to perform calibration and background subtraction. For this, standard procedures in Mantid^23^ were used, resulting in the scattering intensity in absolute units (cm^–1^) against Q (Å^–1^). Small-angle X-ray scattering (SAXS) experiments were conducted at MAX-IV laboratories, Lund, Sweden, on the Co-SAXS beamline. Using a temperature-regulated sample changer, the samples were measured in 1.5 mm outer diameter borosilicate capillaries (Hilgenberg GmbH), over a Q-range of 0.0012–1.5 Å. Data was reduced and background subtracted using ATSAS,^24^ and data analysis was performed using SASview 5.0.6.^25^

For the fluorescence measurements, a Cary Eclipse Fluorescence Spectrometer was used. A Hanna Instruments Karl Fischer Volumetric Titrator-HI903 was used to measure the water content of the neat solvents in triplicate (Table S6). Surface tension measurements were made using the pendant drop method with an FTA1000 Drop Shape Analyzer at 50 °C, to reduce the solution viscosity to enable drops to form. The density of 1:2 and 1:4 CA:Gly (Table S7), used in SLD calculations, was measured at 20 °C using an Anton Paar DMA 4500 M. ^1^H NMR in D_2_O on a 400 MHz Bruker Neo was used to check for thermal stability of the DES, through measurement and comparison of results from before and after heating some of the solvent in a sealed vessel at 120 °C for 24 h (SI Figure S7). No signs of reactions between the components could be seen, but citric acid appeared to decompose after prolonged heating at 120 °C. No signs of significant component decomposition could be seen for the lower temperatures used in the other experiments. Measurements of the complex viscosities for 1:2 and 1:4 CA:Gly (Figure S8) over the temperature range of 20–60 °C were made on an Anton Paar 301 rheometer using a 25 mm diameter cone with a cone angle of 0.973°. A COSMOS-RS model was used to predict the ideal and simulated eutectic ratios for citric acid:glycerol mixtures (Figure S9). Details of the simulation can be found in the Supporting Information.

## Results and Discussion

### Surfactant Solubility

Rasool et al.^26^ determined the eutectic ratio in the citric acid:glycerol system to be around 1:4 CA:Gly, based on visual inspection of the homogeneity of a range of molar ratios. Simulation of the eutectic ratio using COSMO-RS predicts that the eutectic point should occur at a citric acid volume fraction of 0.23, which corroborates that earlier report. We have therefore made detailed studies of self-assembly in solutions at the eutectic ratio, and also at 1:2 citric acid:glycerol, to allow comparison with micellization in 1:2 choline chloride:glycerol.

The solubility of common surfactants, the anionic sodium dodecyl sulfate (SDS) and sodium lauroyl sarcosinate, the cationic C_12_TAB and C_16_TAB, and the nonionic Brij L4 (main component C_12_EO_4_) and Brij L23 (main component C_12_EO_23_), were tested within mixtures based on different ratios of citric acid and glycerol, and, for comparison, neat glycerol was also tested. For a better comparison, surfactants with a 12-carbon containing tail group were chosen, as well as the very common 16-carbon containing tail group equivalent of C_12_TAB, to see any effect of the alkyl chain length. In choline chloride:malonic acid deep eutectic solvent, significant micellar elongation was observed for C_16_TAB, ascribed to electrostatic interactions between the anionic acid and the cationic headgroups.^27^ C_12_TAB micelles, however, remained more globular in the same solvent. Here, we therefore compare the micelles formed by C16 and C12 cationic surfactants in the CA:Gly mixtures, as well as comparing cationic to nonionic surfactants.

The anionic surfactant SDS showed negligible solubility (<0.01 wt %) at room temperature or at 50 °C in 1:2, 1:4, or 1:10 CA:Gly, as well as in neat glycerol, and was deemed insoluble. SDS has been previously noted to form fibrillar aggregates visible by eye in glycerol solutions cooled from 60 to 25 °C, at concentrations as low as 0.8 wt %.^28^ Similarly low solubility for SDS in the DES 1:2 ChCl:Gly has been reported by Matthews et al.,^29^ who observed formation of a lamellar gel phase above 1.9 wt % in this solvent, after initial dissolution at 60 °C. Solutions at 0.1 wt % in 1:2 ChCl:Gly were opaque at room temperatures and polarizing optical microscopy and SANS measurements showed the formation of fractal structures attributed to solid SDS aggregates in these solutions.^29^ Sodium lauroyl sarcosinate was similarly tested and also deemed insoluble (solubility < 0.01 wt %).

The cationic surfactant C_12_TAB, on the other hand, showed good solubility at room temperature at every tested solvent molar ratio as well as in neat glycerol. C_16_TAB also showed good solubility at 50 °C in all solvents but was insoluble at room temperature. Brij L4 and Brij L23 were not significantly soluble (<0.01 wt %) in 1:4 or 1:10 CA:Gly, or in neat glycerol, but showed good solubility (>10 wt %) in 1:2 CA:Gly at room temperature. The cloud point for Brij L4 in 1:2. CA:Gly, however, was around 30 °C, while that for Brij L23 was ca. 50 °C; thus, all further measurements on these surfactants were made at 25 °C. To test the behavior of a nonionic surfactant at an intermediate glycerol concentration, the solubility of Brij L4 was also tested in 1:3 CA:Gly, in which it was also found to be soluble. The influence of the addition of 10 wt % of water to the solvent before the addition of surfactant has also been tested. Brij L4 and Brij L23 remained soluble in 1:2 CA:Gly + 10 wt % water, while they remained insoluble in 1:4 CA:Gly + 10 wt % water. Both the dry and the hydrated 1:2 CA:Gly surfactant solutions remained stable at room temperature for over 6 months. This clearly shows that the change in solubilizing capability is not an effect of, e.g., moisture absorbed from the atmosphere or due to a slow process of phase separation as a result of the higher viscosity of the 1:2 mixture. The change can be clearly attributed to the change in solvent properties stemming from the change in molar ratio of the components. It has been shown for similar DES systems that the addition of up to 50 wt % of water only leads to slight structural changes within the DES, while the main DES-forming interactions remain intact.^30,31^ While solubilization is a complex process, this could be a contribution to why the solubilization behavior remains similar in the dry and hydrated DES, if the specific DES interactions at this molar ratio contribute to the solubility of these nonionic surfactants.

### Critical Micelle Concentration

In Figure 1, the relative fluorescence signal I_1_/I_3_ of pyrene against the surfactant concentration for C_12_TAB and Brij L23 at room temperature and C_16_TAB at 50 °C in 1:2 CA:Gly can be seen, with C_12_TAB in 1:4 CA:Gly and neat glycerol shown in the Supporting Information (SI Figure S1). The first and third peaks in the fluorescence spectrum of pyrene are sensitive to the polarity of the solvent. Measuring the signal as a function of surfactant concentration, the typical sigmoidal behavior characteristic of the formation of micelles can be seen. Below the CMC, pyrene is free in the more polar solution and partitions into the nonpolar core of the micelles above the CMC. The measured CMC values, alongside literature values for water, the ionic liquid ethylammonium nitrate, and other DES, can be seen in Table 1.

**Figure 1:**
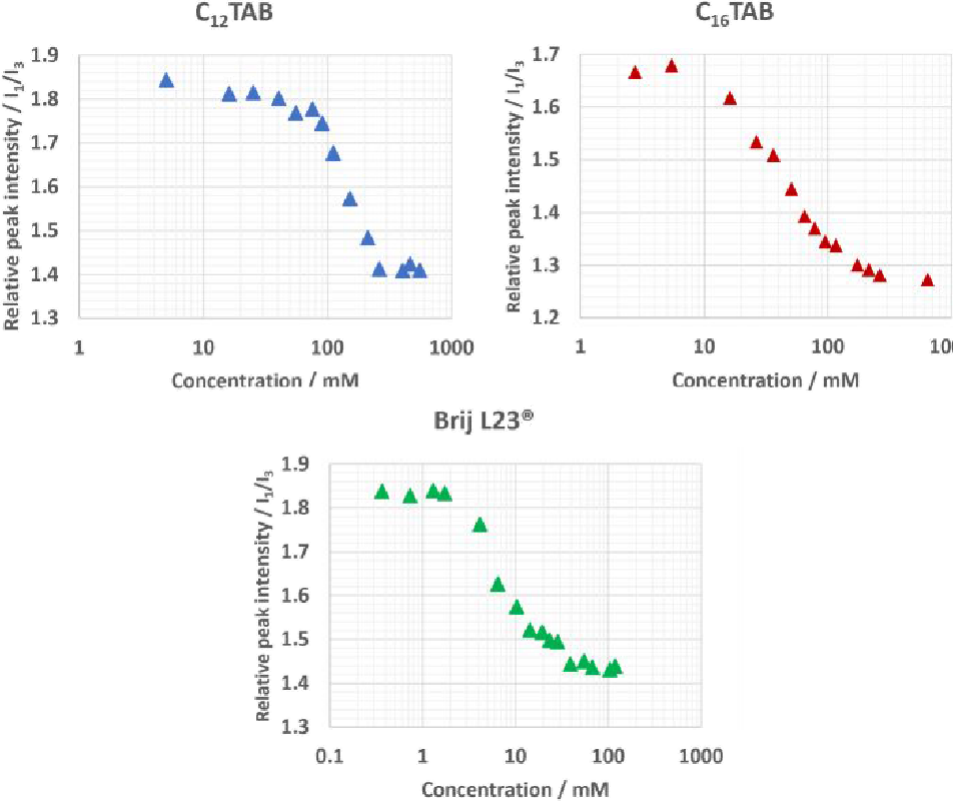
Relative peak intensity of pyrene fluorescence peaks against concentration of C_12_TAB, C_16_TAB, and Brij L23 in 1:2 CA:Gly.

**Table 1:** Surfactant CMC Values in Different Solvents

surfactant	solvent	CMC/mM	temp./ °C
C_12_TANO_3_			
	1:2 CA:Gly	53 ± 9	50
	1:3.5 Ce(NO_3_)_3_·6H_2_O:Urea	5.4 ± 0.9 ^18^	25
C_12_TAB			
	1:2 CA:Gly	71 ± 6	20
	1:4 CA:Gly	82 ± 6	20
	glycerol	77 ± 5	20
	1:2 ChCl:Gly	22 ± 2 ^32^	40
	1:1 ChCl:Mal	54 ± 6 ^27^	50
	1:3.5 Ce(NO_3_)_3_·6H_2_O:Urea	5.4 ± 0.9 ^18^	25
	water	15.9 ^33^	20
	EAN	18 ^34^	50
C_16_TANO_3_			
	1:2 CA:Gly	24 ± 11	50
C_16_TAB			
	1:2 CA:Gly	10 ± 4	50
	1:2 ChCl:Gly	0.9 ± 0.1 ^32^	40
	1:1 ChCl:Mal	1.5 ± 0.3 ^27^	50
	water	1.14 ^35^	45
Brij L23			
	water	1.15 ^36^	35
	1:2 CA:Gly	2.6 ± 0.7	25
	water	0.091 ^37^	20

The CMC of C_12_TAB was found to be 71 ± 6 mM in 1:2 CA:Gly, 82 ± 6 mM in 1:4 CA:Gly, and 77 ± 9 mM in neat glycerol. This is higher than that reported for this amphiphile in 1:1 choline chloride: malonic acid (ChCl:Mal; 54 ± 6 mM)^27^ and other DES,^18,32^ water,^33^ and ethylammonium nitrate (EAN).^34^ C_16_TAB also shows significantly high solubility in 1:2 CA:Gly, having a CMC an order of magnitude higher than that reported in water (10 ± 4 vs 1.14 mM at 45 °C^35^) and in other DES. Although these CMCs have been measured at different temperatures, the CMC values for aqueous solutions of C_12_TAB^33^ and C_16_TAB^35^ do not change much over the temperature range used (less than the uncertainties in our CMC measurements), so the variations in CMC in these polar DES are more likely due to solvent properties rather than temperature variation. Insufficient C_12_TANO_3_ and C_16_TANO_3_ were available to carry out fluorescence experiments, so the CMC for these surfactants in 1:2 CA:Gly was estimated using surface tension via pendant drop analysis, using dilutions of solutions prepared for SAXS measurements (discussed below). The high viscosity of these solutions makes generating reproducible droplets difficult; therefore, these values have large uncertainties. They have similar orders of magnitude to those for the corresponding bromide surfactants in this DES.

The CMC of the nonionic Brij L23 in 1:2 CA:Gly is also an order of magnitude higher than in water (2.6 ± 0.6 vs 0.091 mM^37^). This shows the high overall surfactant solubility in the solvents studied here. The ratio I_1_/I_3_ of the pyrene fluorescence peaks can also be used as an indicator to compare neat solvent polarity, and it can be seen that the polarity follows 1:2 CA:Gly >1:4 CA:Gly > Glycerol (SI Table S1), and all show significantly lower polarity than that reported for 1:2 ChCl:Gly.^38^ This could contribute to the higher surfactant solubility in the glycerol- and citric acid-based systems.

### Small Angle Scattering

Looking to potential future applications of these systems in the templating of catalytic materials, we intended to avoid the presence of halide ions in solution, which can poison catalysts. We therefore investigated the behavior of C_12_TANO_3_, C_16_TANO_3_, Brij L23, and Brij L4 surfactants in 1:2 and 1:4 CA:Gly, as well as in neat glycerol, in systems where visually homogeneous solutions were formed. To avoid the introduction of halide ions into the system, we used cationic surfactants with a nitrate instead of a bromide counterion, which are adjacent in the Hofmeister series and are known to have very similar effects on CMC, micelle size, and morphology for C*_n_* TA^+^ surfactants.^39^ SANS measurements of different concentrations of fully hydrogenated (h-) or deuterated (d-) C_12_TANO_3_ (20 °C) and of C_16_TANO_3_ (50 °C) in 1:2 CA:Gly were taken in different combinations of an isotopically substituted solvent for an overall set of five contrasts. Similarly, hydrogenated Brij L23 and L4 (20 °C) were measured in 1:2 CA:Gly for an overall total of three contrasts. All contrasts are listed in the Supporting Information (SI Table S2b). The scattering length densities (SLD) used for fitting the data are also listed in the Supporting Information (SI Table S2a). Fitting of the neutron data started with the samples of the lowest concentration, which can be expected to show relatively reduced intermicellar interactions. The different contrasts were fitted simultaneously, as well as each contrast individually, to allow for comparisons.

The fitted SANS data can be seen in Figures 2 and 3, as well as in the Supporting Information (SI Figures S2–S4). Prior to model fitting, model independent pair distance distribution function (PDDF) analysis was conducted (SI Figure S5), but due to the presence of intermicellar interactions even at low concentrations, the only conclusion that was drawn from it was that there are no extended structures such as cylinders or disks present in the systems containing C_12_TANO_3_, C_16_TANO_3_ and Brij L23.^40,41^

**Figure 2:**
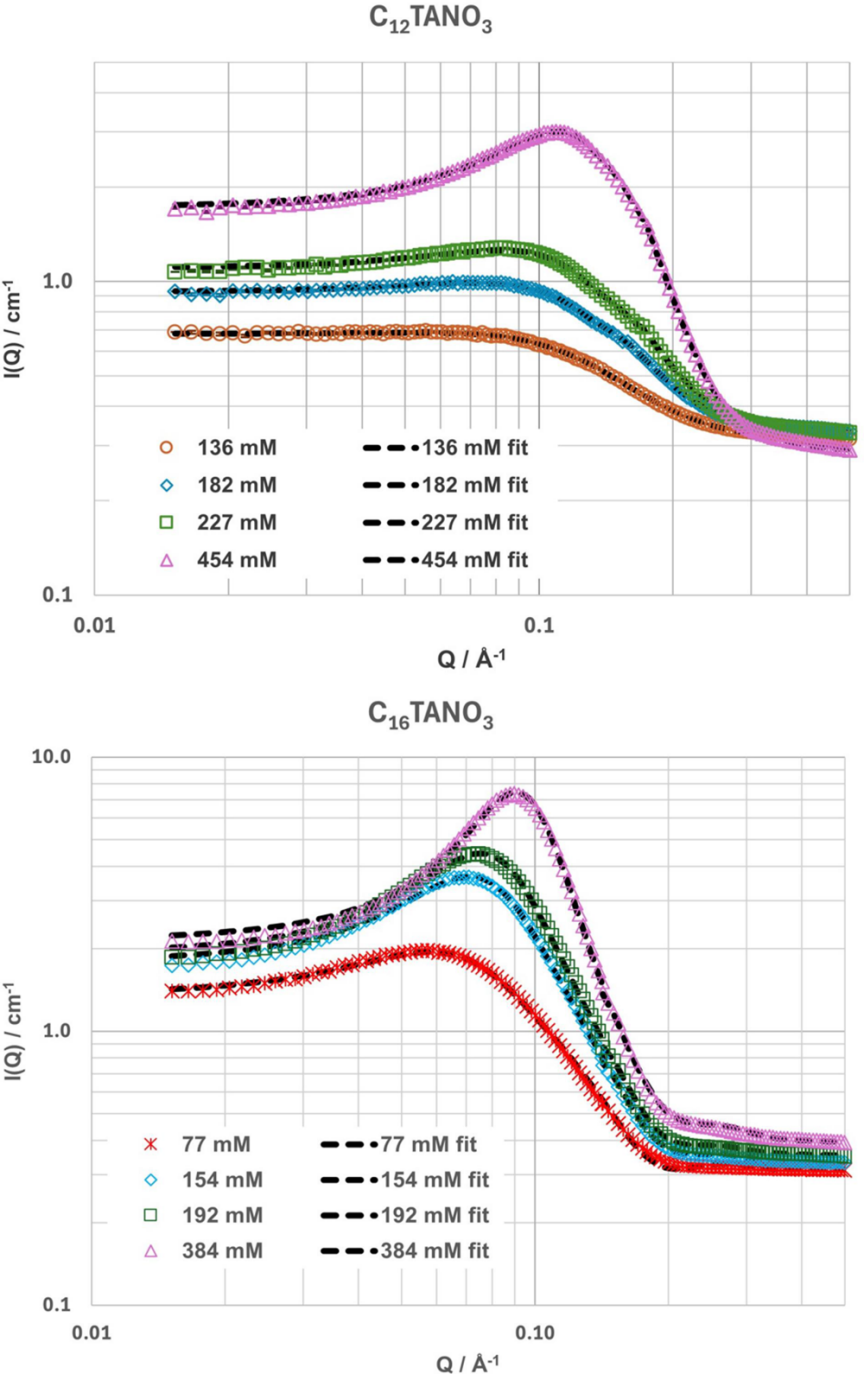
Plots showing SANS data (points) and best fits (dashed lines) of h-C_12_TANO_3_ (top) and h-C_16_TANO_3_ in 1:2 h-CA:d-Gly at different concentrations. Measurements of C_12_TANO_3_ were taken at 20 °C and of C_16_TANO_3_ at 50 °C due to an increased Krafft temperature. Error bars are within the area of the marker size and, therefore, are not included.

**Figure 3:**
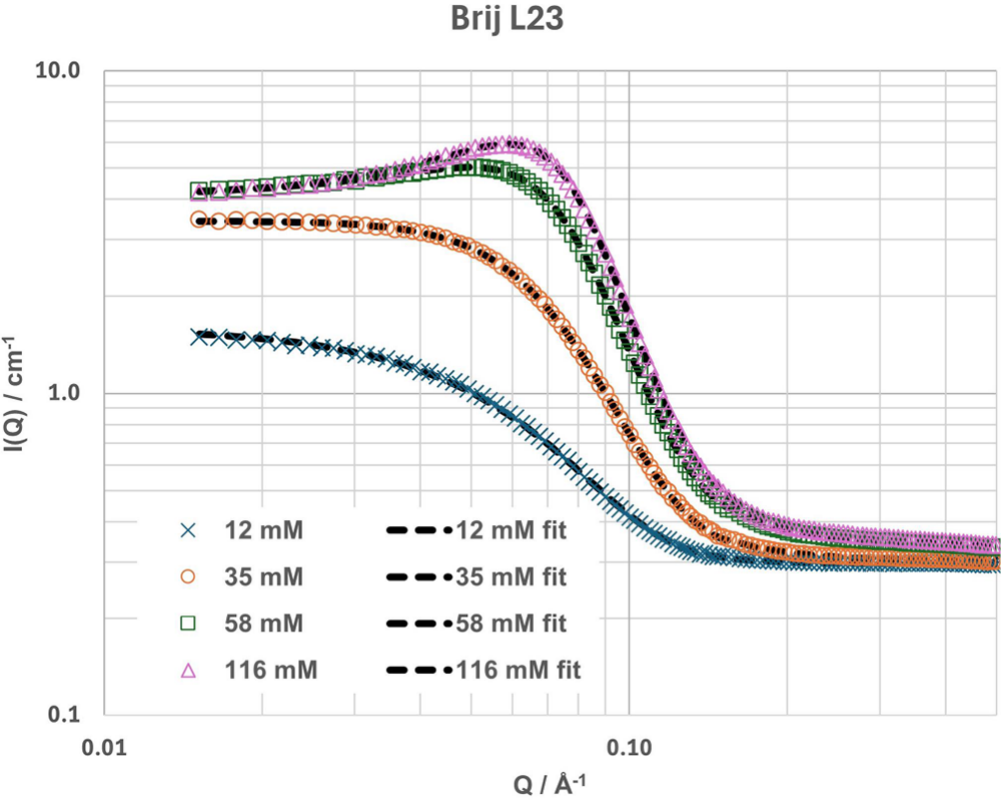
Plots showing SANS data (points) and best fits (dashed lines) of h-Brij L23 in 1:2 h-CA:d-Gly at different concentrations. Measurements were taken at 20 °C. Error bars are within the area of the marker size and are therefore not included.

### Cationic Surfactant Micelle Formation

C_12_TANO_3_ micelles were fit using a simple sphere model with a low, fixed polydispersity (Schulz) of 0.15 (Figure 2, SI Figure S2a–d), where the hydrophobic tail groups formed the core of the micelle. All concentrations required the use of a structure factor to account for the intermicellar interactions. An effective hard sphere structure factor was used, as parameters such as the dielectric constant of the solvent and the real ionic strength of these solutions are unknown. A similar approach has been used for other studies of micelles in DES.^11,27,32^ The structural fit parameters against concentration can be seen in Figure 4 (numerical values listed in SI Tables S3 and S4). The aggregation number *N*_agg_ was calculated using the fitted core volume and the known volume of a surfactant tail group calculated from the Tanford formula.^42^

**Figure 4:**
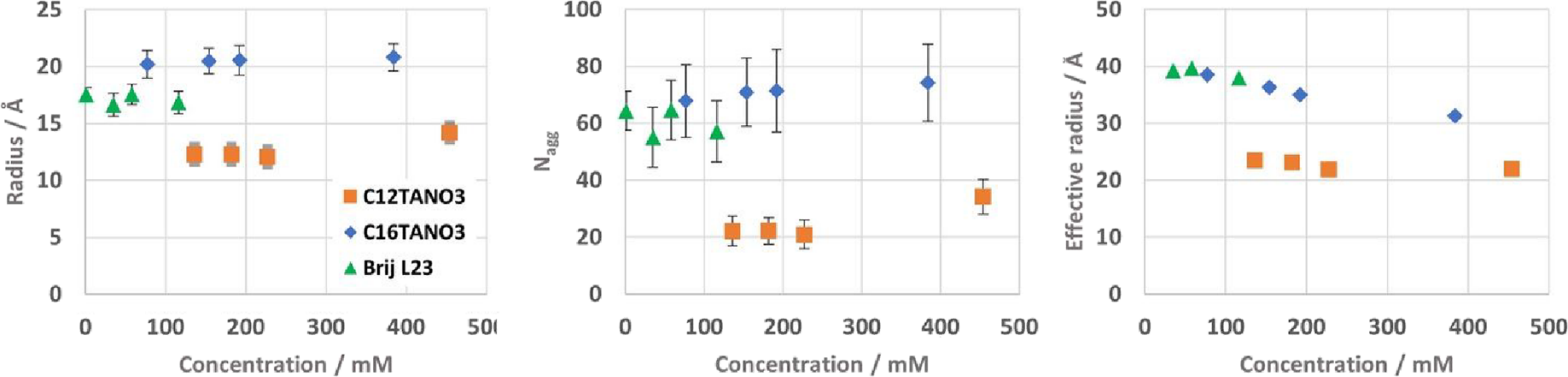
Structural fit parameters against concentration for C_12_TANO_3_ (orange squares), C_16_TANO_3_ (blue diamond), and Brij L23 (green triangles) in 1:2 CA:Gly: radius or core radius (left), aggregation number, *N*_agg_ (middle), effective hard sphere radius for the structure factor, *R*_eff_ (right; uncertainties within the marker size).

In SAS, it is difficult to differentiate a polydisperse sphere from an ellipsoid with a low aspect ratio, as they are mathematically very similar.^43^ An oblate ellipsoidal model with a low aspect ratio (χ = ∼ 0.5), without polydispersity, was also able to fit the data well (parameters listed in SI Table S3). While the equatorial radius is reasonable (∼19 Å), the polar radius (∼8–11 Å) is on the borderline of being unphysically short. The formation of oblate ellipsoidal micelles, as opposed to prolate micelles, would also be contrary to what has been found for C_12_TANO_3_ and C_12_TAB micelles in similar DES, and would require more solid evidence. The aspect ratio also decreases with increasing concentration (3 wt % χ = 0.41, 10 wt % χ = 0.58), which is contrary to the increase in aspect ratio with concentration, which can usually be seen for other ellipsoidal micelle systems.^43–45^ A prolate ellipsoidal model, on the other hand, does not provide a good fit to all contrasts. Therefore, a polydisperse spherical model was chosen.

As has been shown in other DES,^11,18,27,32^ the headgroup can be highly solvated in a DES surfactant system. Analogous to the reported systems, we applied a core–shell sphere model, where we allowed the SLD of the headgroup to fit within the constraint of the calculated headgroup SLD and the solvent SLD, while the SLD of the tail group remained fixed. However, in our case, this led to a very high volume fraction of solvent in the shell of around 95%, indicating the lack of sufficient contrast to model. It also led to a very small core radius (∼10 Å), which would lead to an unphysically small micelle with an aggregation number *N*_agg_ of ∼ 12 molecules in a micelle, alongside a shell that is unreasonably large for headgroup/headgroup+counterions size (∼11 Å). This led us to reject a core–shell model and use a uniform sphere model, based solely on the micelle core.

The core radii at all four measured concentrations (12.1 ± 1.3 to 14.2 ± 1.2 Å) are smaller than the length of a fully extended C_12_ tail group (16.8 Å).^45^ This is similar to that found in the ionic liquid, ethylammonium nitrate (EAN) for C_12_TAB (15.0 Å) and C_12_TANO_3_ (11.0 Å),^46^ and slightly smaller than what has been found in water for C_12_TAB at low concentrations (16.4 Å)^47^ and the DES 1:3.5 cerium nitrate hexahydrate: urea (Ce­(NO_3_)_3_:U) for C_12_TAB (15.0 ± 1.1 Å) and C_12_TANO_3_ (16.2 ± 0.9 Å),^18^ indicating a significant amount of tail coiling within the micelle core (a table summarizing micelle dimensions in these systems can be seen in SI Table S3c). A low degree of solvent penetration into the palisade layer of the micelle, as occurs in aqueous systems, especially for smaller spherical micelles,^48,49^ can also contribute to the micelles appearing smaller. For C_12_TAB in more similar DES systems such as 1:2 choline chloride: glycerol (ChCl:Gly) or 1:1 choline chloride: malonic acid (ChCl:Mal) the micelles were fit to ellipsoidal models with an aspect ratio of <2 (14.8 ± 0.3 Å with χ = 1.64 and 14.6 ± 0.6 Å with χ = 1.9 respectively),^27,32^ which as mentioned above, can be treated like a polydisperse sphere. SAXS data for C_12_TAB in 1:4 CA:Gly can also be fitted to very similar parameters as the SANS data for C_12_TANO_3_ (see Table S5 and Figure S6). This indicates similarities between these systems and a degree of independence from the CMC, as the CMC in CA:Gly is significantly higher than in ChCl:Mal and ChCl:Gly (Table 1). As C_12_TAB in water forms ellipsoidal micelles with higher aspect ratios at higher concentrations,^50^ both the formation of spheres or ellipsoids is reasonable, and transitions between them, depending on the solvent components and ratios, surfactant solubility, and interactions in the solvent, as well as temperature and concentration, can be expected. Interestingly, a significantly higher structure factor contribution to the SANS scattering pattern can be seen in the data when comparing the CA:Gly system to both ChC:Gly and ChCl:Mal. This indicates that the charge screening effect in our system is significantly reduced, probably due to the lack of ChCl or equivalent ions in the solution, which is in agreement with the lower polarity of the CA:Gly system indicated by the fluorescence measurements. Some charge screening can still be expected. While measurement of the degree of component deprotonation is not as straightforward as in water, other studies have shown acidity in ChCl:dicarboxylic acid-based DES.^51,52^ A simple test using pH paper also indicated significant acidity in our system (pH ∼ 1).

For the lower three surfactant concentrations, the radius remains almost identical, while it increases for the highest concentration (*N*_agg_ 22 vs 34), which is double the third concentration, while the polydispersity remains stable around 0.15. This indicates an increase in the number of surfactant molecules per micelle, or potentially a contribution from a shift to more ellipsoidal micelles. A decrease in the effective interaction distance in the structure factor (*R*_eff_) with increasing concentration can also be seen, which is expected as the micelles start interacting more due to their concentration-induced closer proximity. As in the similar systems mentioned above, this shows that the micelle structure is only slightly affected by the change in concentration, in this range.

The C_16_TANO_3_ containing samples were similarly fit using the same model (Figure 2 and SI Figure S3a–d), and the structural fit parameters against concentration can also be seen in Figure 4 (numerical values listed in SI Table S3). Similar to C_12_TANO_3_, it was also possible to fit the SANS data using a low aspect ratio ellipsoidal model, but in this case, a prolate ellipsoidal model could be fit well to every contrast, providing reasonable values (parameters listed in SI Table S3). However, as the aspect ratio remained almost identical over the whole concentration range, the polydisperse spherical model was chosen again. This was a simple sphere model, as fitting a core–shell model resulted in similar concerns as for C_12_TANO_3_.

The radius of the core at all concentrations (∼20.5 Å) is slightly below that of the fully extended C_16_ chain (21.7 Å), indicating a small amount of tail coiling or solvent penetration. It is smaller than what has been found for C_16_TAB in water (21.8 Å)^53^ and larger than what has been found for C_16_TAB in EAN (19.2 Å).^54^ While C_16_TAB in 1:2 ChCl:Gly shows comparable spheroid micelles, C_16_TAB micelles in 1:1 ChCl:Mal are significantly elongated (aspect ratio χ > 10). The authors hypothesized that this change in surfactant packing behavior for the 1:1 ChCl:Mal system in comparison to 1:2 ChCl:Gly, could be due to the interaction of a potentially deprotonated carboxylic acid with the surfactant headgroup. This was based on a similar behavior seen in aqueous systems, where it has been shown that C_16_TAB forms spherical to ellipsoidal micelles at low to moderate concentrations,^50,53^ while the introduction of acids and salts can lead to a change in the surfactant packing behavior, forming elongated structures due to screening of the charge on the headgroups by small oppositely charged species.^55,56^ It is therefore interesting to see that no such effect can be seen in our system, which should also contain significantly deprotonated carboxylic acid molecules, and interactions of the surfactant headgroup with these can be expected. Malonic acid and citric acid also have similar p*K*_a_s (p*K*_a_1 2.8 and pKa2 5.7 in Mal, p*K*_a_ 2.79 in CA),^57,58^ indicating similar dissociation behavior in an aqueous environment. However, these solvents are not an aqueous environment, and the degree of dissociation is also dependent on the other components. As mentioned in a previous section, in our system, the CMC is about an order of magnitude higher than that in 1:1 ChCl:Mal (10 vs 1.5 mM). Therefore, the concentration of micelles will be lower in our system at a similar surfactant molarity, as more of the surfactant can remain free in solution. However, even comparing the lowest concentration (38 mM) used by Sanchez-Fernandez et al.^27^ to our highest concentration (384 mM), no similarities can be seen. As was shown for 1:1 ChCl:Mal, it is primarily the acid that penetrates into the micelle headgroup shell, and a steric effect of the significantly larger citric acid can be expected, potentially leading to spherical packing of the surfactants being more energetically favorable here. Another difference between the systems is the absence of charged choline and chloride ions, which can lead to a change in micellar interaction behavior. Similar to what can be seen with C_12_TANO_3_, a stronger contribution of the structure factor can be seen in our SANS data, in the form of a more pronounced interaction peak. Therefore, similar to aqueous systems, where the addition of salts leads to the elongation of the micellar structures, here the absence of ChCl could have an effect opposed to the effect of the carboxylic acid deprotonation, as the degree of charge screening is decreased in comparison to the ChCl-based systems.

Micelles formed by C_16_TANO_3_ do not show a significantly different behavior from C_12_TANO_3_ micelles, besides being larger and having relatively fewer coiled tail groups or less solvent penetration. As can be seen in C_12_TANO_3_, the effect of the concentration on the structures formed by C_16_TANO_3_ is relatively small. The radius increases slightly with concentration (20.2–20.8 Å), while *R*_eff_ decreases, and the polydispersity remains stable at 0.15.

### Nonionic Surfactant Self-Assembly

The SANS data and fits for the nonionic Brij L23 (main component C_12_EO_23_) are shown in Figure 3 (additional contrasts in SI Figure S4). The data were fitted using a core–shell spherical model with a polydispersity (Schulz) of around 0.25. At the lowest concentration (12 mM), no structure factor was used, with a hard sphere structure factor being used for higher concentrations, and the structural fit parameters can be seen in Figures 4 and 5 (numerical values listed in SI Table S4). Brij L23 has a degree of chain length variation, and for the SLD calculation, C_12_EO_23_ was assumed, as small variations in chain length do not have a big impact on the SLD, but a larger degree of polydispersity of the micelles can be expected.

**Figure 5:**
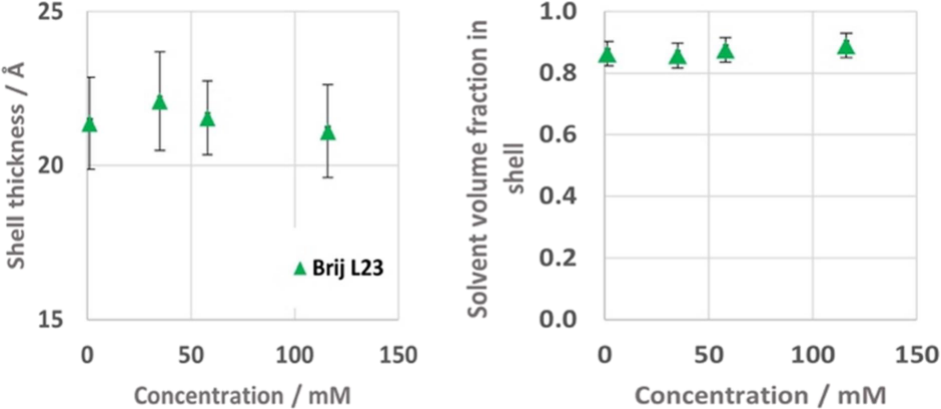
Core–shell model specific structural fit parameters against the concentration of Brij L23 in 1:2 CA:Gly: shell thickness (left), solvent volume fraction in shell (left).

A fit of the system without a core–shell model is possible, but gives significantly worse fits for some contrasts. In the fits without the core–shell model, the core radii at all concentrations are large in comparison to the expected C_12_ tail length (16.8 Å^45^), with a radius of ∼24 Å, and an *N*_agg_ of ∼165 molecules, which is unreasonable for a spherical micelle unless there is penetration of headgroups or solvent into the core. As this effect cannot be seen with the other micelles, solvent penetration seems to be less likely. Some penetration of the headgroups into the core can be expected, as small angle studies for similar systems (C_12_EO*_n_*) in water, the 1:3.5 cerium nitrate: urea DES and EAN have found cores with radii from around the extended C_12_ tail length to slightly larger (up to 20 Å), with some dependence on the temperature and concentration.^18,59–63^ However, for these literature systems and fits, core–shell models were used as well. In our case, the fitted radius of ∼24 Å would indicate a very high degree of headgroup penetration; the fitted parameters are listed in the Supporting Information (Table S4). This is likely if the headgroup has low solubility in the solvent, but considering the higher CMC in comparison to water, it seems unlikely that it would be significantly less soluble than that in water. This, together with the worse fit of the model without a shell, led us to choose the core–shell sphere model. Here, fitting the SLD in a core–shell model also shows a lower degree of solvent penetration in the shell (∼85%) in comparison to the cationic surfactants discussed above, potentially due to charge or steric effects, which allows for sufficient contrast to fit a shell.

Like the cationic micelles, the Brij L23 micelles can be fitted well to an ellipsoidal model with a low axial ratio (prolate, χ = ∼ 1.7), and the dimensions of the ellipsoidal micelle are acceptable (equatorial radius = ∼14–17 Å) (SI Table S4). However, here, again, the lack of a significant increase in the aspect ratio with increasing concentration leads to the choice of a polydisperse spherical model over an ellipsoidal one.

The radius and shell thickness from the spherical core–shell model (Figures 4, 5 and SI Table 3a) are in good agreement with what has been found in the literature for this surfactant in water.^59,61–63^ Unlike what can be seen under some temperature and concentration conditions in water, and for C_12_EO*_n_* in the 1:3.5 cerium nitrate: urea DES and EAN,^18,60,64^ the shell thickness here (∼21.5 Å) indicates that the headgroups are fairly extended. This is more similar to that found in water at lower concentrations (24.7,^61^ 22.1 Å^63^). A reduction in shell thickness can usually be attributed to either a loss of contrast toward the edge of the shell, as the relative amount of solvent increases, or to coiling of the headgroup chain induced by lower headgroup solubility due to the high ionic content in the solvent.^18,64^ Better solubility of the headgroup in our DES with fewer ionic components could therefore contribute to the headgroup being more extended.

Comparing the studied concentrations, no significant changes in radius, shell thickness, or solvent volume fraction can be seen in this concentration range, while the expected decrease in *R*_eff_ as concentration increases, can be observed.

Brij L4 forms larger structures (SI Figure S4c) over the measured concentration range, which cannot be fit using models such as simple cylinders or lamellae. There is also an absence of peaks indicating the formation of crystalline structures, such as liquid crystalline phases. The slope at low-Q (around 0.01 Å^–1^, log–log scale) is close to −2, which could indicate the presence of disordered lamellae or sponge phases, but shows variation with concentration.^65^ Many ethylene oxide-based surfactants show a rich phase behavior in water, with a significant likelihood of multiple phases coexisting, especially for those surfactants with smaller headgroups.^66–68^ The data show a diffuse liquid peak in the lower Q-region, which is typical for disordered lamellar structures, whereas L_α_ lamellar phases tend to exhibit a better-defined, sharper peak.^65,67^ The position of the broad bump moves to higher Q as the volume fraction of the surfactant increases, indicating that the structure becomes more compact as surfactant is added, but no further improvement of ordering is seen.

### Influence of Solvent Composition on Cationic Micelle Formation

To compare the effect of the solvent composition on the micelle structure, SAXS measurements (MAX IV, Lund, Sweden) of C_12_TANO_3_ (20 °C) and C_16_TANO_3_ (50 °C) in 1:2 CA:Gly, 1:4 CA:Gly, and neat glycerol were taken. C_12_TAB (20 °C) was also measured in 1:2 CA:Gly and 1:4 CA:Gly (SAXS fits: SI Figure S6, fitting parameters: SI Table S5). To fit the data in 1:2 CA:Gly, the same models as for the neutron data were used, using the parameters of the lowest concentration as starting values in the SAXS fits.

C_12_TANO_3_, C_12_TAB, and C_16_TANO_3_ data can all be fitted well to a spherical model with hard sphere interactions and 0.2 polydispersity (Schulz) in these solvents. The C_12_TANO_3_ micelles in 1:2 CA:Gly are slightly bigger than in the other two solvents (13.4 ± 1.0 Å at 227 mM vs 11.6 ± 1.0 Å at 205 mM in glycerol). However, as they are within the error of each and similar in magnitude to the size difference between the SANS (12.3 ± 0.9 Å) and SAXS results in 1:2 CA:Gly, this indicates that there are no significant differences in micelle morphology in these three systems. Similarly, C_16_TANO_3_ micelles show no significant differences in radius or polydispersity in all three solvents. This shows that while the surfactant solubility varies between the different solvents, within this concentration range, they all form similar micellar structures. This is not unexpected, as micelles of C_12_TAB and C_16_TAB in water also form spherical micelles at lower concentrations and only elongate at higher concentrations or upon the addition of, e.g., salts, as discussed above. Due to the difference in surfactant solubilities between the systems, it can be expected that, similar to the CMC, transitions into other self-assembled structures, such as lamellae or liquid crystalline phases, will also be shifted and potentially altered.^69^

## Conclusions

Here we have presented the solubilities and self-assembled structures of different surfactants in citric acid:glycerol-based solvent systems. We have shown that the solubility of the cationic surfactants C_12_TANO_3_ and C_16_TANO_3_ is similar in glycerol, 1:4 CA:Gly, and 1:2 CA:Gly, and the CMC of the bromide analogues of these surfactants, C_12_TAB and C_16_TAB in 1:2 CA:Gly, have similar magnitudes to those of the nitrate surfactants. By adjusting the molar ratio, it is also possible to dissolve the nonionic surfactants Brij L23 and Brij L4, in 1:2 CA:Gly, but not in neat glycerol or CA:Gly at a 1:4 molar ratio. Here, Brij L23 forms spheroid micelles, while Brij L4 forms more disordered phases, such as sponge phases or mixed phases. The solubility of these nonionic surfactants is interesting, as many EO-based surfactants are not soluble in common choline chloride-based DES, like ChCl:Urea and ChCl:Glycerol. This solubility and ability to form micelles are retained upon the addition of 10 wt % of water to 1:2 CA:Gly, opening up a wider use range due to a significant decrease in viscosity caused by water addition. On the other hand, Brij L23 and Brij L4 remain insoluble in neat glycerol or 1:4 CA:Gly even upon the addition of water. The CMC of all three surfactants (measured with a bromide counterion on the cationic surfactants) in 1:2 CA:Gly is significantly above that found in water, in 1:1 choline chloride: malonic acid,^27^ and 1:2 choline chloride: glycerol.^32^ Similar to what has been reported for some carboxylic acid-containing DES,^51,52^ we expect CA in our system to be significantly deprotonated (pH paper test indicates a pH of ∼1). However, comparing the peak ratio I_1_/I_3_ from pyrene fluorescence for the neat solvents, it can be seen that the solvents studied here have a significantly lower polarity than choline chloride-based systems, potentially contributing to the higher solubility of the surfactants.

SANS and SAXS studies were also performed, revealing the formation of slightly polydisperse spheroid micelles for all three micelle-forming surfactants in 1:2 CA:Gly. For C_12_TANO_3_ and C_12_TAB, this is similar to what has been found in other DES. For C_16_TANO_3_, it is similar to micelles formed in 1:2 ChCl:Gly,^32^ but different from those in 1:1 ChCl:Mal, where significantly elongated micelles are formed.^27^ This is interesting, as for the ChCl:Mal system, Sanchez-Fernandez et al. hypothesized that deprotonated malonic acid could contribute to elongated micelles becoming favorable by screening charges on the cationic surfactant headgroups,^27^ but that effect cannot be seen in our system. In their system, choline chloride and malonic acid could potentially synergistically shield charges better than just deprotonated citric acid in our system. A steric effect of the significantly larger citric acid, in comparison to malonic acid, altering its penetration behavior into the micellar shell and the relative charge densities could also play a role. An effect of the difference in counterion binding around the micelle headgroups vs in the bulk solvent can also be expected, as counterions have been shown to influence micellization behavior in DES.^70^

In general, no significant changes with increasing concentrations besides the expected increase in intermicellar interactions could be seen in our system, showing no signs of an increasing micelle aspect ratio within the measured concentration range. The C_12_TANO_3_, C_12_TAB, and C_16_TANO_3_ micelles in 1:2 and 1:4 CA:Gly, as well as in neat glycerol, all have comparable spheroidal micelles.

The nonionic Brij L23 micelles can be fitted using a spherical core–shell model, revealing cores and shells with extended chains more alike to what can be found in water, than for similar C_12_EO*_n_* surfactants in a 1:3.5 cerium nitrate:urea DES^18^ or EAN,^64^ where the shells tend to be thin, indicating more headgroup coiling or solvent penetration.

Overall, our studies shed light on the effect of varying the solvent composition on surfactant solubility in mixtures such as deep eutectic solvents as well as the effect of component choice on micelle structures. Untangling the interactions responsible for these variations will enhance our ability to rationally select suitable components and compositions for specific applications, hopefully opening up the full potential of these liquids in the future.

## Supplementary Material



## Data Availability

Raw small angle neutron scattering data is available at Institut Laue-Langevin (ILL): Grenoble; DOI:10.5291/ILLDATA.9-10-1674.

## Notes

Ion exchange procedure for surfactants; fluorescence methods and data; SANS and SAXS data analysis information and data; water content in the solvents; and thermal stability, viscosity, density, and eutectic point simulation for the solvents (PDF)
