# Supplementary material for: Cationic and Nonionic Surfactant Micelles in a Halogen-Free Carboxylic Acid-Based Deep Eutectic Solvent
Source: Langmuir. 2025 May 13;41(20):12489–98. doi: 10.1021/acs.langmuir.4c05370 (PMC12120992; doi:10.1021/acs.langmuir.4c05370)
Supplement: Supplementary file 1 [file la4c05370_si_001.pdf]

Supplementary information:

Cationic and Non-ionic Surfactant Micelles in a Halogen-Free Carboxylic Acid-Based Deep Eutectic Solvent

Elly K. Bathke[a], Sylvain Prévost[b], Fátima Herranz-Trillo[c], Subramee Sarkar[a], Laura Deeming[a], Ronak Kakadiya[a], Maggie Kroon[a], Daniel T. Bowron[d], Karen J. Edler[a]

[a]Centre for Analysis and Synthesis, Department of Chemistry, Lund University, Naturvetarvägen 22, Lund, 223 62, Sweden
[b]Institut Laue-Langevin, 71 avenue des Martyrs, Grenoble, 38042, France
[c]MAX IV Laboratory, Fotongatan 2, Lund, 22484, Sweden
[d]ISIS Neutron and Muon Source, Science and Technology Facilities Council, Rutherford Appleton Laboratory, Didcot, OX11 0QX, Oxfordshire, United Kingdom

E-mail:
karen.edler@chem.lu.se

Contents

	Page
Ion exchange of surfactants	S1
Fluorescence	S1
SANS data analysis	S1
Extra SANS data	S4
PDDF Analysis	S10
SANS model fitting	S10
SAXS data and model fitting	S12
Water content	S13
Thermal stability of the solvent	S14
Rheological measurements	S14
Eutectic behaviour of CA:Gly	S15
Density of CA:Gly	S15
References	S15

Ion exchange of surfactants

Initially the ion exchange resin Amberlite® IRN-78 (OH-form) was prepared by stirring it in a >1M aqueous NaOH solution for at least 3 hours. The resin was repeatedly rinsed with ultrapure water until the eluent reached a pH of 7. The surfactant was dissolved in water and the exchange took place in a fresh batch of resin at least three times, forming the surfactant hydroxide. The final eluent was tested for the presence of halogens by taking an aliquot and adding silver nitrate. Concentrated nitric acid was then added until the solution reached a pH of ~6. Finally, the solution was frozen and dry surfactant powder generated by use of lyophilization.

Fluorescence

For fluorescence measurements a known concentration pf pyrene was dissolved in ethanol and diluted down to a concentration of 10 µg/mL. 0.05 mL of the pyrene solution was added to an empty vial, and the ethanol evaporated. 5 g of the solvent with surfactant solution was added, and stirred at 50°C for at least 12 hours for the cationic surfactant samples and stirred for at least 24 hours at room temperature for the non-ionic surfactant samples.
The samples were measured using an excitation wavelength of 337 nm, an excitation slit opening of 5 and an emission slit opening of 2.5, over a wavelength spectrum of 350 to 500 nm.

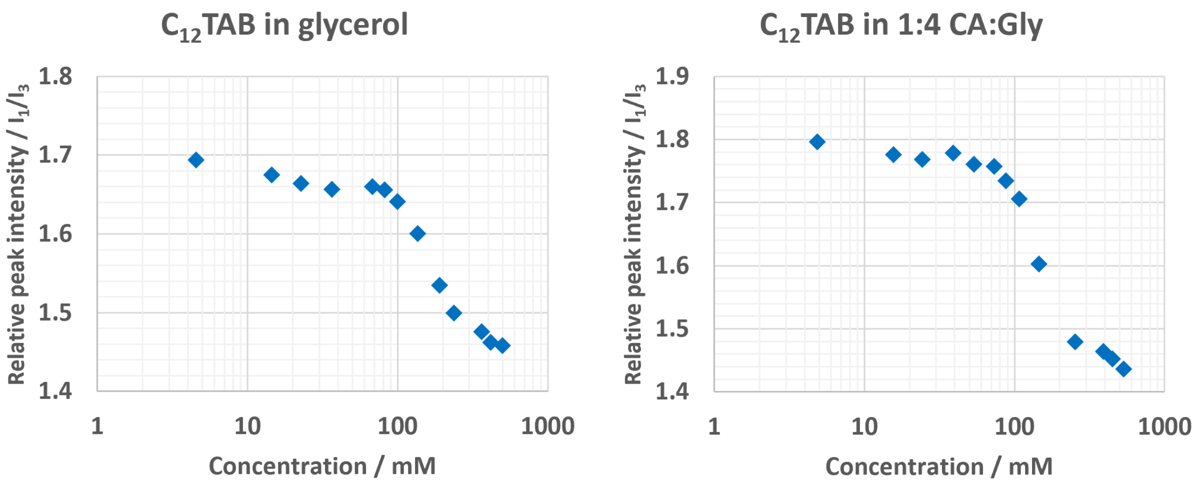

Fig. S1: Relative peak intensity of pyrene fluorescence peaks against concentration of C_{12}TAB in 1:4 CA:Gly and neat glycerol.

	I_1/I_3
Glycerol	1.68
1:4 CA:Gly	1.83
1:2 CA:Gly	1.86

Table S1 : The ratio of the intensity of the first and third fluorescence peak of pyrene in the different solvents, measured at 20°C.

SANS data analysis

The scattering data was analyzed using a polydisperse sphere or polydisperse core shell sphere model combined with a hard sphere structure factor, as implemented in SASView 5.0.6.[1] The local monodisperse approximation was used to calculate the scattered intensity I(Q), i.e.:

$$I(Q)=scale(V_f/V)P(Q)S(Q)+background \qquad (S1)$$

Where V_f is the volume fraction of the material, V is the particle volume, P(Q) is the form factor, S(Q) is the structure factor.
The form factor for a sphere[2] is given by P(Q) = $\langle F(Q)^2\rangle$ where

$$F(Q) = \left[3V(\rho_s - \rho_{solv})\frac{\sin(Qr)-Qr\cos(Qr)}{(Qr)^3}\right] \qquad (S2)$$

Where V is the volume of the sphere, r is the radius of the sphere, ρ_s is the scattering length density of the sphere, ρ_{solv}, is the scattering length density of the solvent. A Schulz polydispersity distribution was applied to the radius.

The form factor for a core shell sphere[2] is given by P(Q) = $\langle F(Q)^2\rangle$ where

$$F(Q) = \frac{3}{V_s}\left[V_c(\rho_c - \rho_s)\frac{\sin(Qr_c)-Qr_c\cos(Qr_c)}{(Qr_c)^3} + V_s(\rho_s - \rho_{solv})\frac{\sin(Qr_s)-Qr_c\cos(Qr_s)}{(Qr_s)^3}\right] \qquad (S3)$$

where V_s is the volume of the whole particle, V_c is the volume of the core, r_s is the radius of the particle, r_c is the radius of the core, ρ_c is the scattering length density of the core, ρ_s is the scattering length density of the shell, ρ_{solv}, is the scattering length density of the solvent. A Schulz polydispersity distribution was applied to the core radius, and the shell radius was the sum of the shell thickness plus the core radius.

A hard sphere structure factor with the Percus-Yevick closure[3] was used to describe the interparticle interactions due to the unknown dielectric constant for the mixtures studied, and also because in these high salt concentration solutions any charge on the micelle is expected to be screened over very short distances, so that the micelles effectively behave as uncharged hard spheres at the low volume fractions used. The exact ionic strength of the solvent is also difficult to estimate as the degree of deprotonation of the acid species is unknown, given the very low amounts of water present (see Table S6). This approach has been used previously for similar solutions of micelles in deep eutectic solvents and although it does not provide a direct physical interpretation of the intermicellar contribution we believe that it is a good approximation which allows the deconvolution of the inter- and intra-particle contributions to the scattering.[4, 5]

Other models were trialed during the fitting process as described in the main manuscript text. Detailed descriptions of these can be found in the SASView Model documentation which can be found online. (https://www.sasview.org/docs/user/qtgui/Perspectives/Fitting/models/index.html)

Table S2 a): Neutron and X-ray scattering length densities for the relevant parts of the system.

	**Neutron SLD (×10^{-6} Å^{-2})**	**X-ray SLD (×10^{-6} Å^{-2})**
1:2 h:h CA:Gly	1.46	15.4
1:2 d:h CA:Gly	2.39	
1:2 d:d CA:Gly	6.11	
1:2 h:d CA:Gly	5.18	
1:4 CA:Gly		12.0
Gly		11.7
h-Trimethylammonium head group	0.180	
d-Trimethylammonium head group	7.12	
h-EO_{23} head group	5.19	10.7
h-C_{12} tail	-0.460	7.30
d-C_{12} tail	6.72	
h-C_{16} tail	-0.430	7.40
d-C_{16} tail	6.81	

Table S2 b): Neutron sample contrasts in 1:2 CA:Gly.

Solvent	Surfactant
(d:h)	$C_{12}TANO_3$ (h)
(h:d)	$C_{12}TANO_3$ (h)
(d:d)	$C_{12}TANO_3$ (h)
(h:h)	$C_{12}TANO_3$ (d)
(d:h)	$C_{12}TANO_3$ (d)
(d:h)	$C_{16}TANO_3$ (h)
(d:d)	$C_{16}TANO_3$ (h)
(h:d)	$C_{16}TANO_3$ (h)
(d:h)	$C_{16}TANO_3$ (d)
(h:h)	$C_{16}TANO_3$ (d)
(d:h)	Brij L23® (h)
(d:d)	Brij L23® (h)
(h:d)	Brij L23® (h)
(d:h)	Brij L4® (h)
(d:d)	Brij L4® (h)
(h:d)	Brij L4® (h)

Extra SANS data

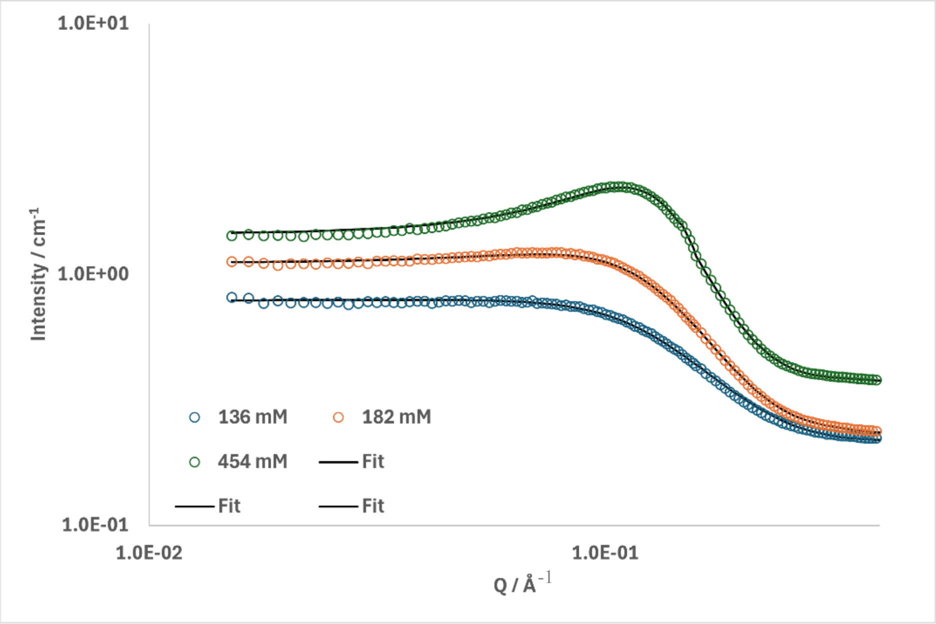

Fig. S2 a): Additional SANS data and simultaneous fits of h-$C_{12}TANO_3$ in 1:2 d-CA:d-Gly.

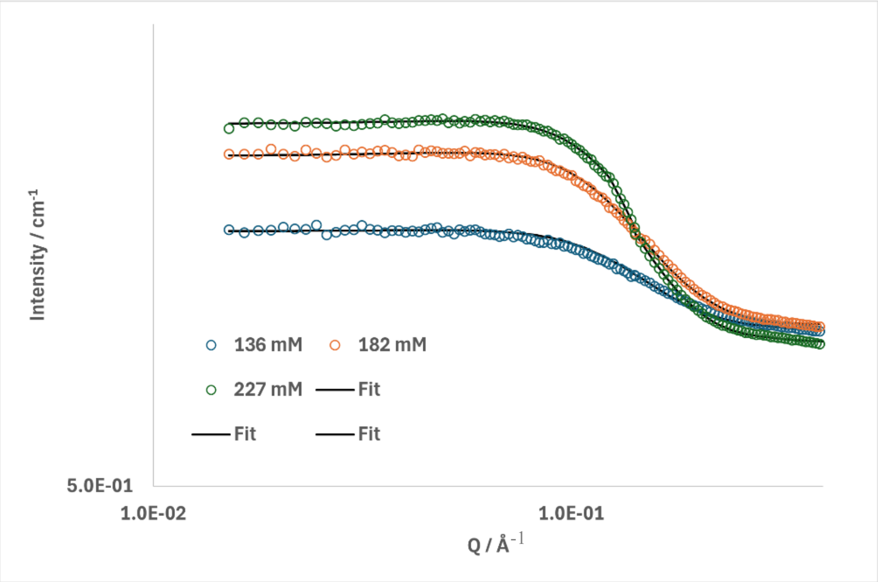

Fig. S2 b): Additional SANS data and simultaneous fits of h-$C_{12}TANO_3$ in 1:2 h-CA:d-Gly.

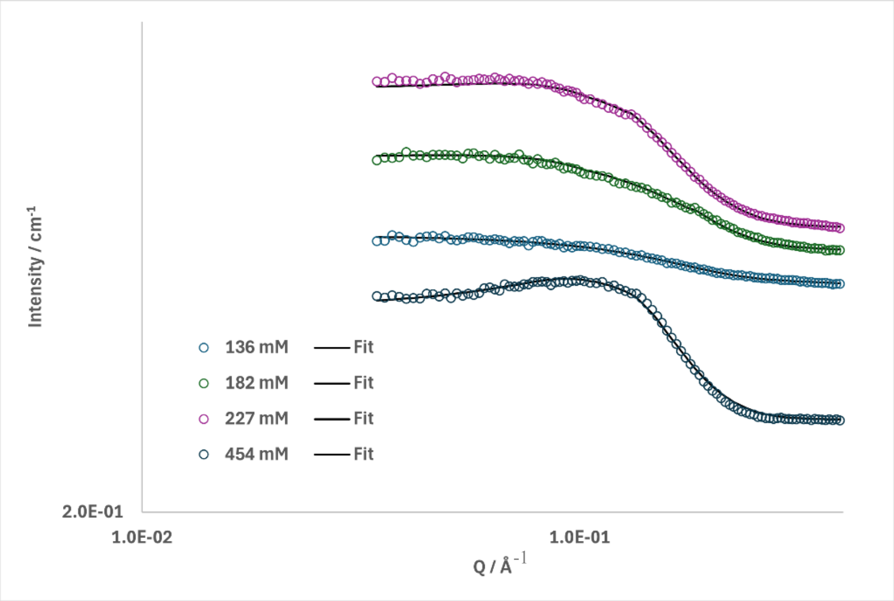

Fig. S2 c): Additional SANS data and simultaneous fits of d-$C_{12}TANO_3$ in 1:2 d-CA:h-Gly.

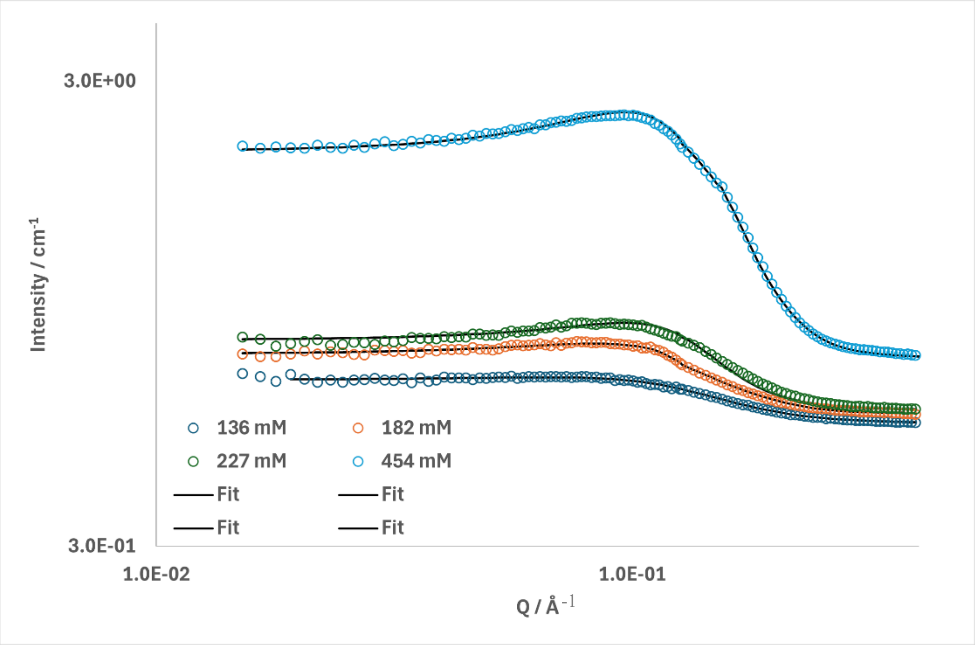

Fig. S2 d): Additional SANS data and simultaneous fits of d-$C_{12}TANO_3$ in 1:2 h-CA:h-Gly.

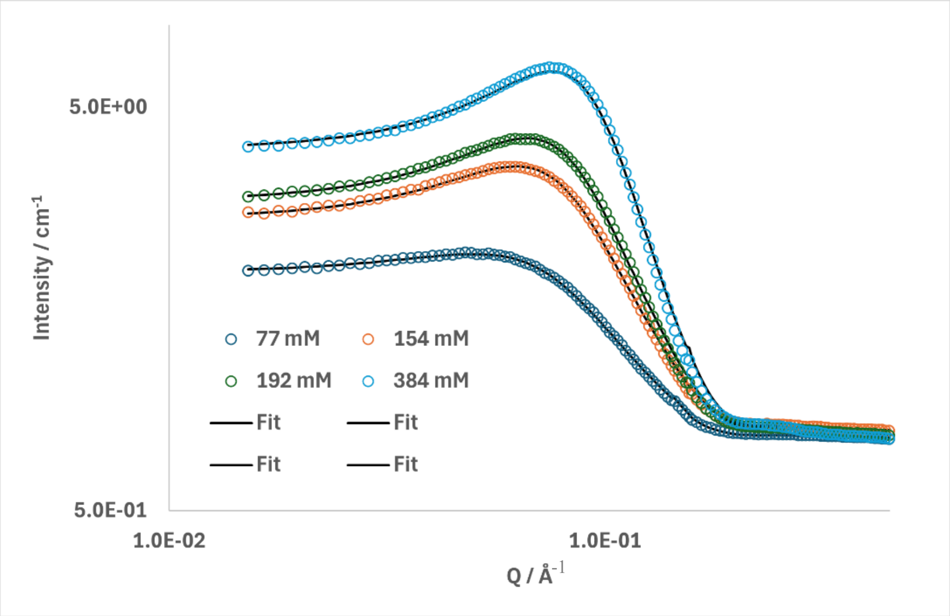

Fig. S3 a): Additional SANS data and simultaneous fits of d-$C_{16}TANO_3$ in 1:2 h-CA:h-Gly.

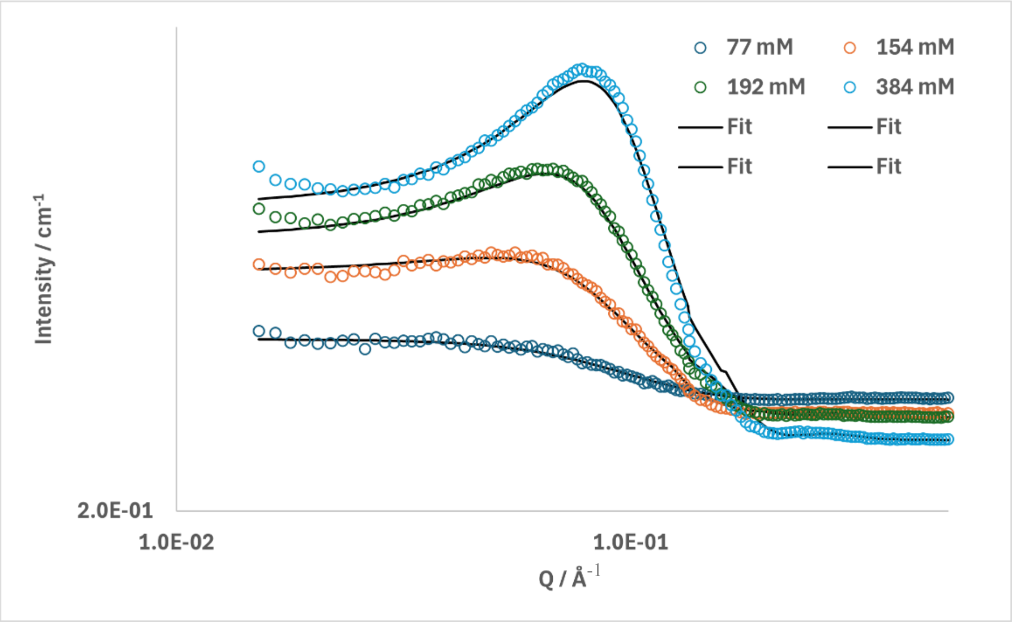

Fig. S3 b): Additional SANS data and simultaneous fits of d-$C_{16}TANO_3$ in 1:2 d-CA:h-Gly.

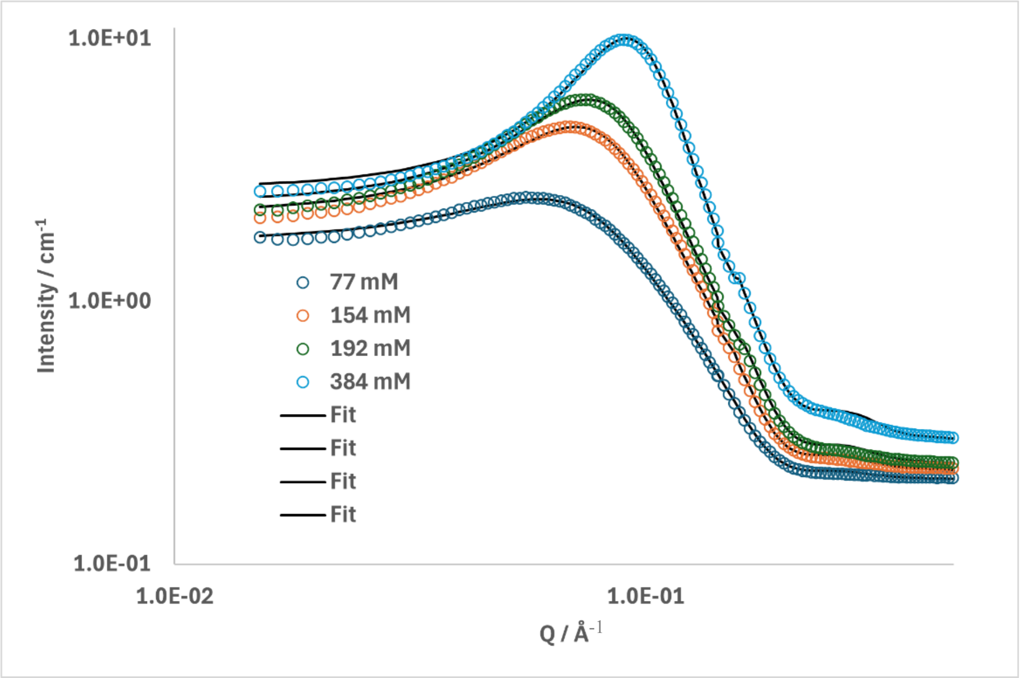

Fig. S3 c): Additional SANS data and simultaneous fits of h-$C_{16}TANO_3$ in 1:2 d-CA:d-Gly.

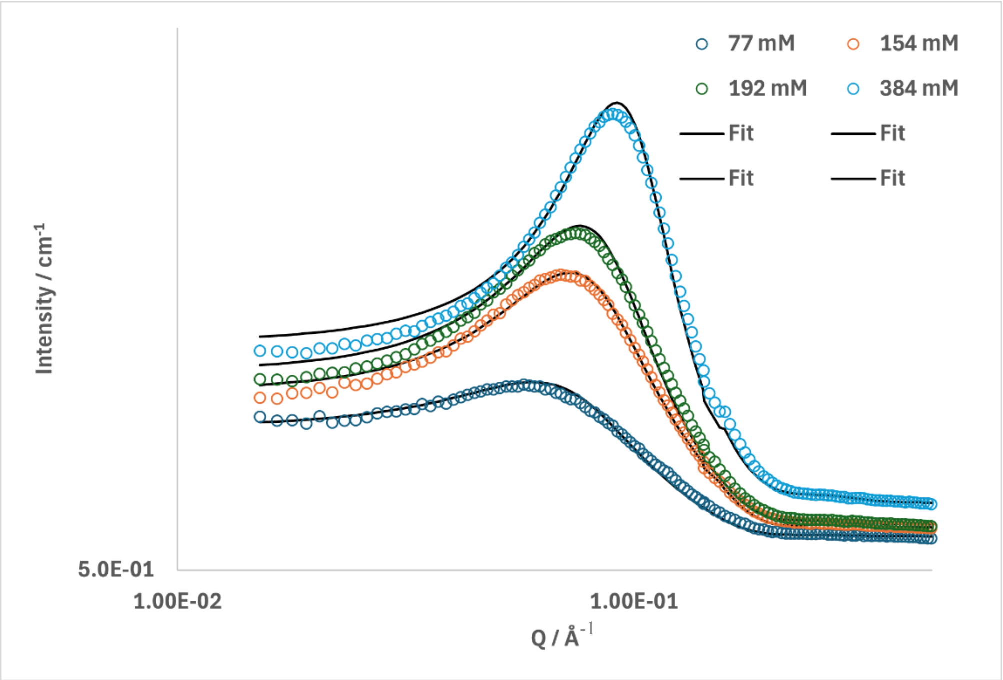

Fig. S3 d): Additional SANS data and simultaneous fits of h-$C_{16}TANO_3$ in 1:2 h-CA:d-Gly.

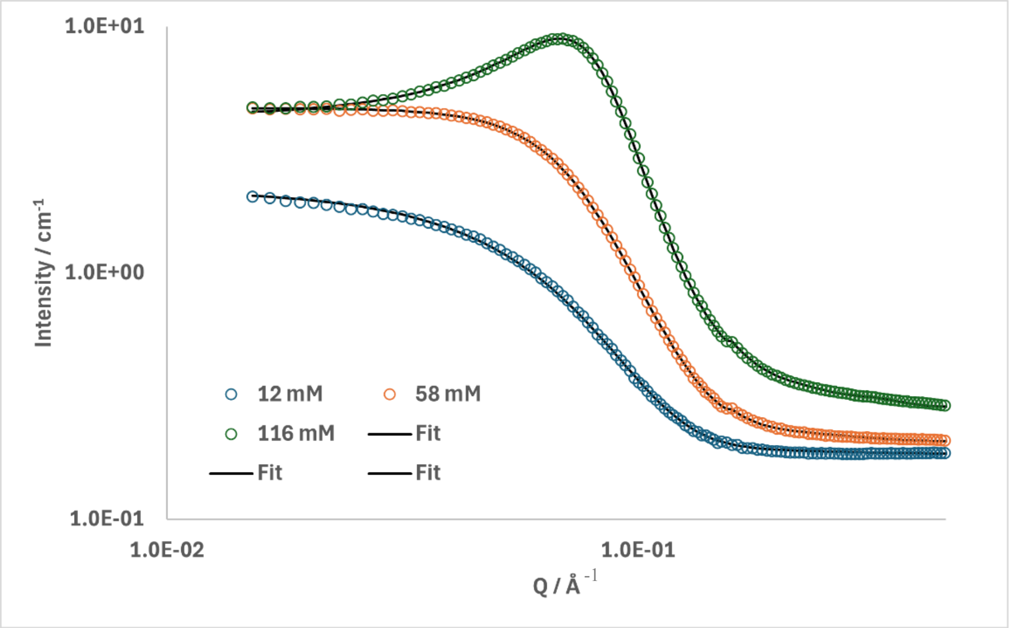

Fig. S4 a): Additional SANS data and simultaneous fits of h-Brij L23® in 1:2 d-CA:d-Gly.

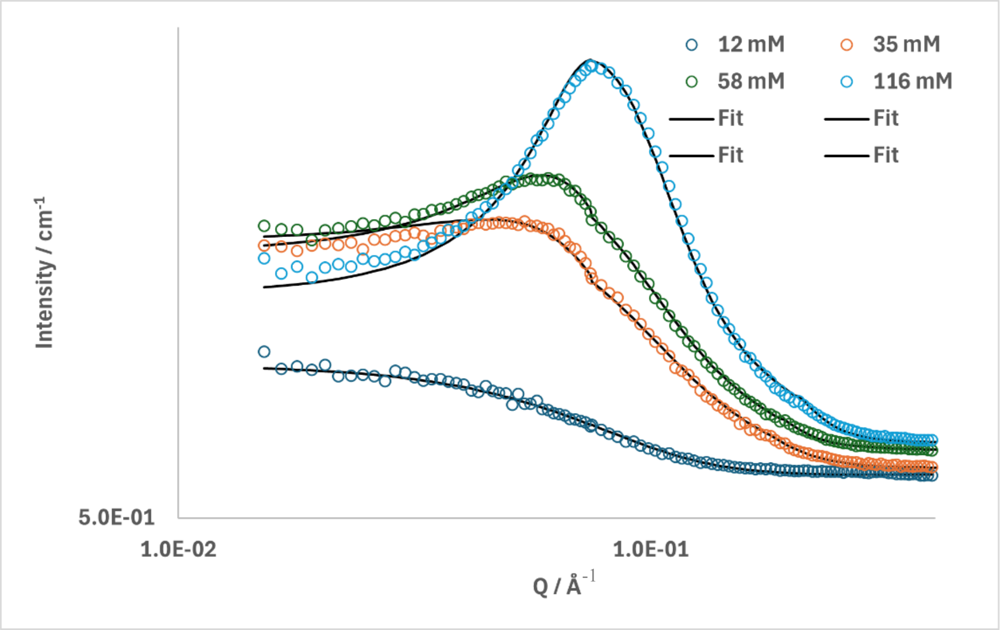

Fig. S4 b): Additional SANS data and simultaneous fits of h-Brij L23® in 1:2 h-CA:d-Gly.

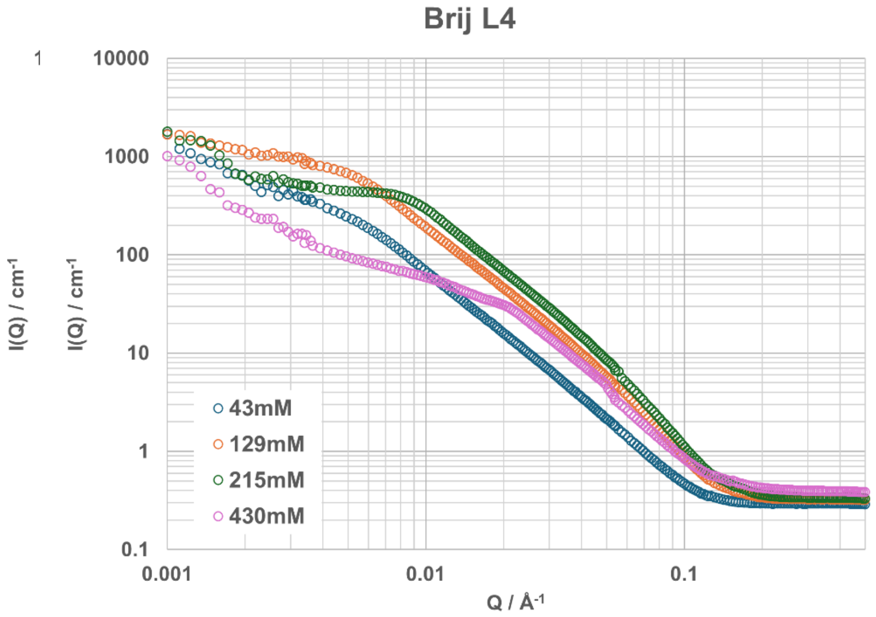

Fig. S4 c): SANS data of h-Brij L4® in 1:2 h-CA:d-Gly.

PDDF analysis

Due to particle interactions even at low concentrations, only limited information can be gained from performing the model independent Indirect Fourier Transformation analysis[6, 7].

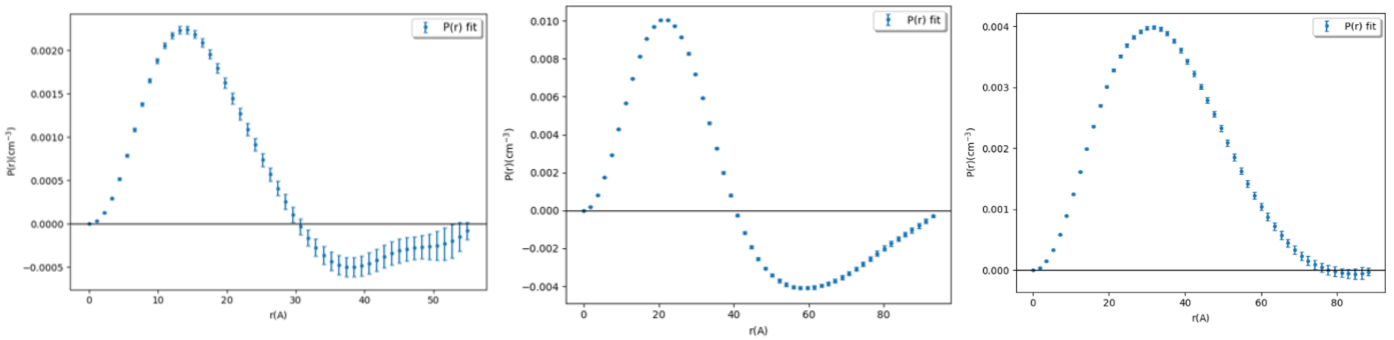

Fig. S5: Representative pair distance distribution functions (PDDF) for $C_{12}TANO_3$ (3 wt%) (left), $C_{16}TANO_3$ (2 wt%) (middle) and Brij L23 (right).

SANS model fitting

Table S3 a): Spherical form factor fit parameters for $C_{12}TANO_3$ and $C_{16}TANO_3$, hard sphere structure factor, 0.15 (Schulz) polydispersity.

**Surfactant**	**Concentration / mM**	**Concentration / wt%**	**radius / Å**	**R_{eff} / Å**	**N_{agg}**
$C_{12}TANO_3$					
	136	3	12.3 ± 0.9	23.5± 1.5	22.15 ± 5.25
	182	4	12.3 ± 0.8	23.1± 1.4	22.20 ± 4.63
	227	5	12.1 ± 0.9	21.9± 1.2	20.93 ± 5.09
	454	10	14.2 ± 0.8	22.0± 1.6	34.18 ± 6.11
$C_{16}TANO_3$					
	77	2	20.2 ± 1.2	38.5± 1.8	67.81 ± 12.82
	154	4	20.5 ± 1.1	36.3± 1.7	70.88 ± 12.04
	192	5	20.5 ± 1.3	35.1± 1.7	71.40 ± 14.43
	384	10	20.8 ± 1.2	31.4± 2.0	74.25 ± 13.60

Table S3 b): Ellipsoid form factor fit parameters for $C_{12}TANO_3$ and $C_{16}TANO_3$, hard sphere structure factor, without polydispersity.

**Surfactant**	**Concentration / mM**	**Concentration / wt%**	**Equatorial radius / Å**	**Polar radius / Å**	**R_{eff} / Å**	**N_{agg}**	**Axial ratio X**
$C_{12}TANO_3$							
	136	3	18.8	7.8	22.5	33.0	0.41
	182	4	19.0	9.5	23.3	41.1	0.50
	227	5	19.2	9.8	21.9	43.0	0.51
	454	10	19.4	11.3	22.2	50.9	0.58
$C_{16}TANO_3$							
	77	2	19.1	29.5	35.1	136.8	1.54
	154	4	19.3	29.7	36.1	140.0	1.54
	192	5	19.4	29.7	35.2	141.2	1.53
	384	10	19.5	30.7	31.4	150.8	1.57

Table S3 c): Best fit values in comparison to literature values of $C_{12}TAB$ and $C_{16}TAB$ in different solvent systems to $C_{12}TANO_3$ and $C_{16}TANO_3$ in 1:2 CA:Gly.

Surfactant	Concentration	Solvent	R or R_{equ} / Å	Axial ratio X
$C_{12}TANO$	136 mM / 3 wt%	1:2 CA:Gly	12.3± 0.9	-
$C_{12}TAB$				
	1 wt%	1:3.5 CeNO:U [8]	15.0	-
	1 wt%	EAN [9]	15.0	-
	156 mM	1:1 ChCl:Mal [10]	14.6	1.9
	43.5 mM	1:2 ChCl:Gly [4]	14.8	1.64
$C_{16}TANO$	77 mM / 2 wt%	1:2 CA:Gly	20.2± 1.2	-
$C_{16}TAB$				
	1 wt%	EAN [11]	19.2	-
	36.3 mM	1:1 ChCl:Mal [10]	19.6	19.6
	38.1 mM	1:2 ChCl:Gly [4]	19.4	12.5

Table S4 a): Sphere form factor fit parameters for Brij L23 with the core shell model, hard sphere structure factor for all besides the lowest concentration, 0.25 (Schulz) polydispersity.

Surfactant	Concentration / mM	Concentration / wt%	Radius / Å	Shell thickness / Å	R_{eff} / Å	N_{agg}	Solvent fraction in shell
Brij L23							
	1.2	1	17.5 ± 0.6	21.37 ± 1.5	-	64.32 ± 6.84	0.86 ± 0.04
	35	3	16.63 ± 1.0	22.09 ± 1.6	39.22 ± 2.6	55.01 ± 10.53	0.86 ± 0.04
	58	5	17.55 ± 0.9	21.55 ± 1.2	39.66 ± 2.3	64.66 ± 10.47	0.87 ± 0.04
	116	10	16.84 ± 1.0	21.11 ± 1.5	38.02 ± 2.0	57.12 ± 10.79	0.89 ± 0.04

Table S4 b): Solid sphere form factor fit parameters for Brij L23, hard sphere structure factor for all besides the lowest concentration, 0.25 (Schulz) polydispersity.

Surfactant	Concentration / mM	Concentration / wt%	Radius / Å	R_{eff} / Å	N_{agg}
Brij L23®					
	1.2	1	24.8	-	182.44
	35	3	24.2	34.5	169.52
	58	5	23.7	38.6	159.23
	116	10	25.3	38.0	193.70

Table S4 c): Ellipsoidal form factor fit parameters for Brij L23 with the core shell model, hard sphere structure factor for all besides the lowest concentration, no polydispersity.

Surfactant	Concentration / mM	Concentration / wt%	Equatorial radius / Å	Polar radius / Å	Shell thickness / Å	R_{eff} / Å	N_{agg}	X_{core}
Brij L23®								
	1.2	1	17.1	29.9	20.1	-	107.65	1.8
	35	3	15.6	29.7	20.8	41.0	86.28	1.9
	58	5	15.4	28.6	20.4	40.1	83.00	1.9
	116	10	14.9	26.2	19.8	38.3	71.22	1.8

Table S5: Spherical form factor fit parameters for $C_{12}TAB$, $C_{12}TANO_3$ and $C_{16}TANO_3$, hard sphere structure factor, 0.2 (Schulz) polydispersity for SAXS measurements.

Solvent	Surfactant	Concentration / mM	Concentration / wt%	Radius / Å	R_{eff} / Å
1:2 CA:Gly	$C_{12}TANO_3$				
		227	5	13.4 ± 1.0	24.0 ± 1.2
		454	10	12.3 ± 1.1	21.1 ± 1.5
	$C_{12}TAB$	227	5	11.3 ± 1.5	21.1 ± 2.0
1:4 CA:Gly	$C_{12}TANO_3$	212	5	11.2 ± 1.2	21.2 ± 1.5
		424	10	11.8 ± 0.9	20.7 ± 1.1
	$C_{12}TAB$	212	5	11.6 ± 1.3	19.8 ± 1.6
Gly	$C_{12}TANO_3$	205	5	11.6 ± 1.0	22.0 ± 1.3
		409	10	12.1 ± 1.1	21.1 ± 1.3
1:2 CA:Gly	$C_{16}TANO_3$	39	1	17.4 ± 1.4	38.1 ± 2.1
		192	5	17.9 ± 1.3	33.4 ± 1.8
		384	10	17.8 ± 1.4	31.2 ± 1.7
1:4 CA:Gly	$C_{16}TANO_3$	35	1	17.6 ± 1.5	45.7 ± 2.2
		177	5	17.4 ± 1.6	34.4 ± 1.9
		354	10	17.2 ± 1.4	30.3 ± 1.7
Gly	$C_{16}TANO_3$	35	1	17.5 ± 1.3	37.8 ± 1.8
		173	5	17.5 ± 1.4	35.0 ± 1.9
		346	10	17.7 ± 1.3	35.0 ± 1.8

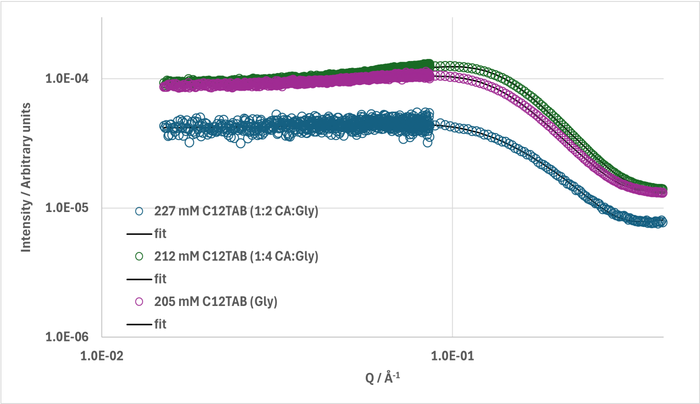

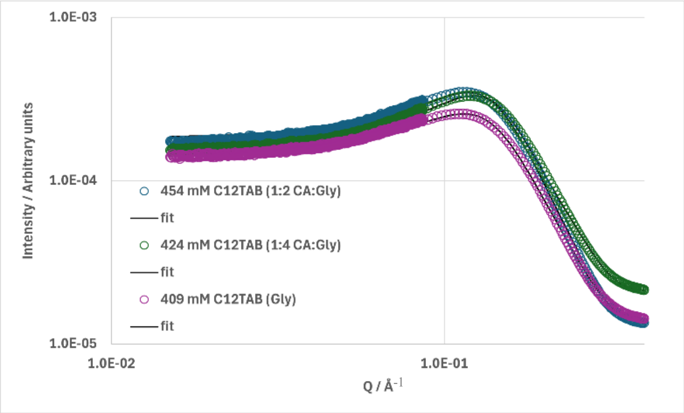

Fig. S6 a): SAXS data and fits of $C_{12}TANO_3$ in 1:2, 1:4 CA:Gly and glycerol: lower (right) and higher (left) concentration.

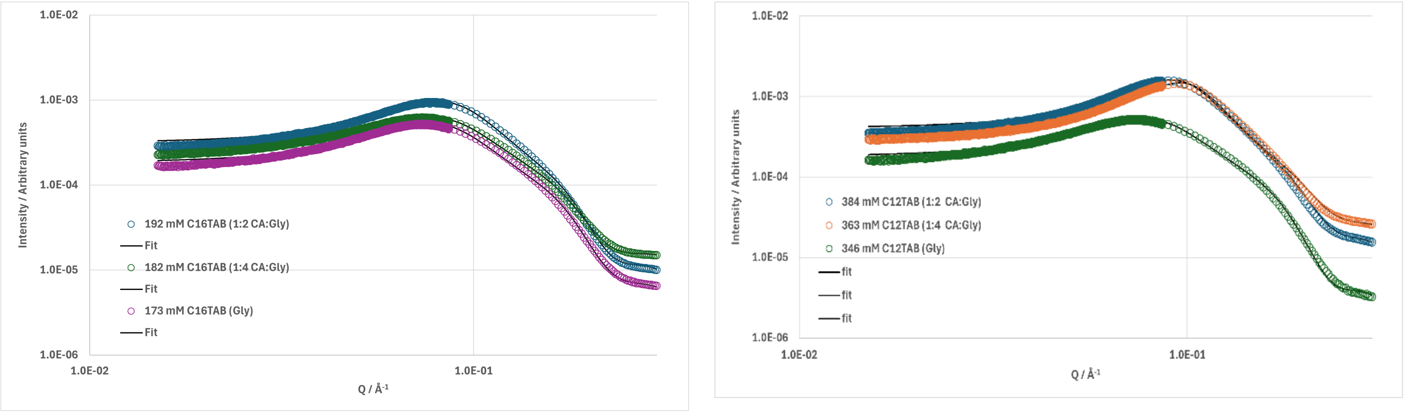

Fig. S6 b): SAXS data and fits of $C_{16}TANO_3$ in 1:2, 1:4 CA:Gly and glycerol: lower (right) and higher (left) concentration.

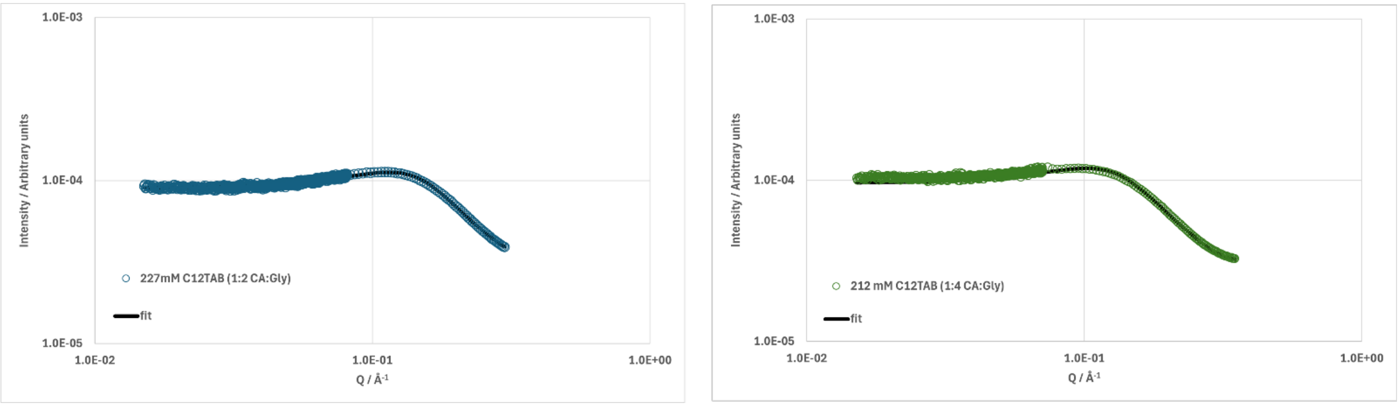

Fig. S6 c): SAXS data and fits of $C_{12}TAB$ in 1:2 (left) 1:4 CA:Gly (right).

Water content

Water content of the different solvents was measured using a Hanna Instruments Karl Fischer Volumetric Titrator HI903. The presence of small acids interferes with the accuracy of the measurement, and for this imidazole was added to neutralize the sample, and the resulting water content subtracted from the measured result. It is still likely that the water content is overestimated from this measurement.

Table S6: Solvent water content.

**Sample**	**Measurement**	**Water content in weight percent**
1:2 CA:Gly		
	1	3.6
	2	3.3
	3	3.8
1:4 CA:Gly		
	1	2.7
	2	2.6
Glycerol		
	1	0.066
	2	0.086
	3	0.081

Thermal stability of the solvent

To test thermal stability ^{1}H-NMR measurements were taken of 1:4 CA:Gly in D_2O on a 400 MHz Bruker Neo before and after heating at 120°C for 24 hours in a closed vessel. No signs of components reacting can be seen, but citric acid seems to degrade.

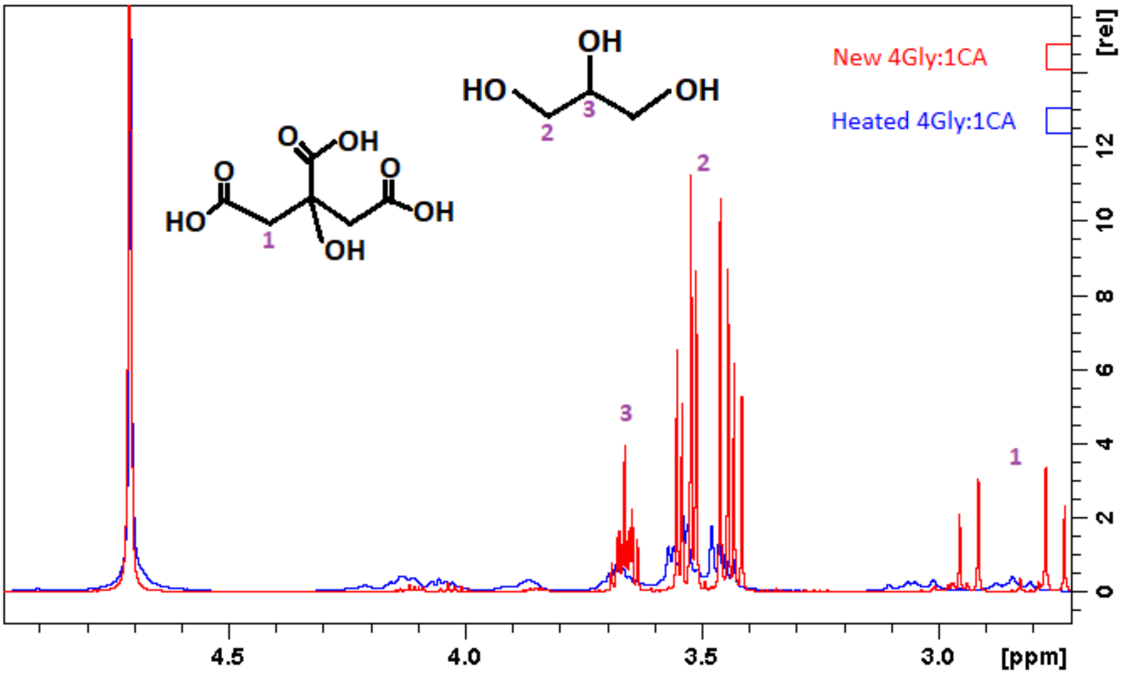

Fig. S7: [1]NMR spectra taken of 1:4 CA:Gly in D_2O of the newly made mixtures vs. the spectra after heating at 120°C for 24 hours.

Rheological measurements

The complex viscosity of 1:2 and 1:4 CA:Gly in dependence on temperature was measured (S8) (conditions)

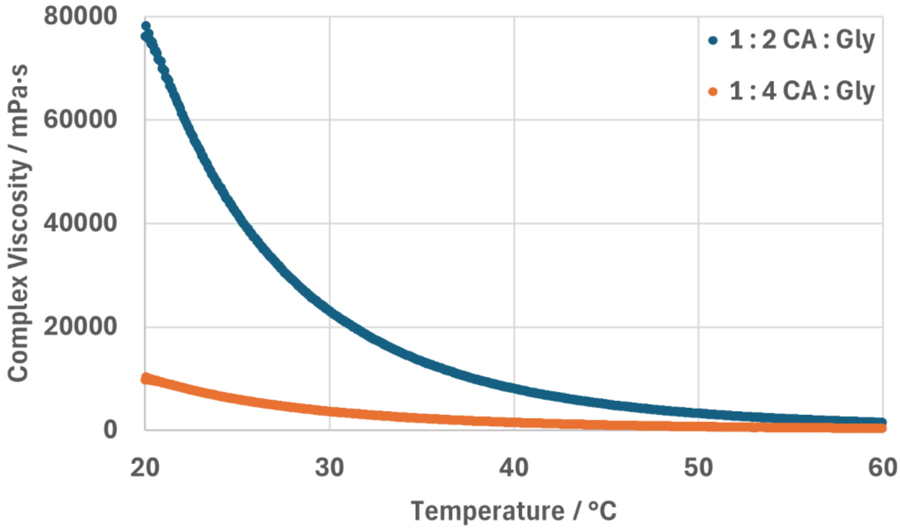

Fig. S8: The complex viscosity of 1:2 and 1:4 CA:Gly in dependence on temperature.

Eutectic behaviour of CA:Gly

COSMO-RS modelling (Figure S9) predicts that the ideal eutectic point is at x(CA) = 0.035, while the COSMO-RS prediction places the eutectic point at x(CA) = 0.230, so close to the ratio of 1:4 CA:Gly.
The citric acid - glycerol solid-liquid equilibrium (SLE) phase diagram was estimated using an open source COSMO-RS implementation[12] and compared to an ideal SLE, computed using thermochemical data publicly available from NIST (Figure S10).[13] Components used in the COSMO-RS calculations underwent energy and RMSD based conformer filtering, and subsequent optimization routines in gas-phase and using a CPCM. The lowest energy conformers were used in the COSMO-RS DFT calculations, which used BP86 functional and def2-TZVPD basis set. All calculations were performed with ORCA 6.0.[14] A complete outline of the methods used and steps involved can be found in e.g. reference [15].

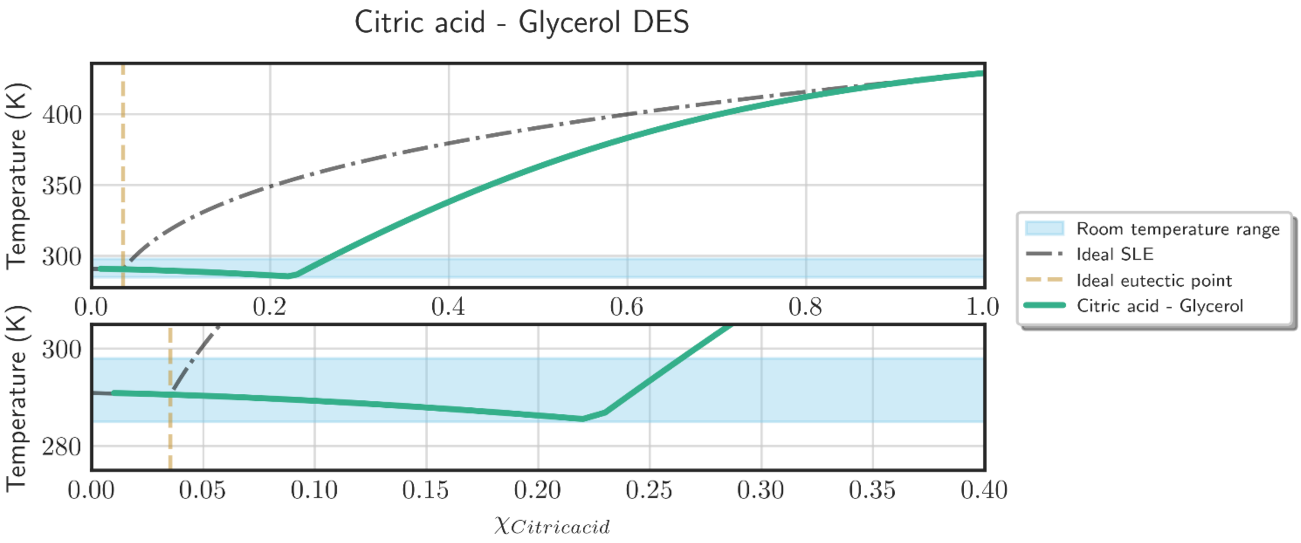

Fig. S9: COSMO-RS prediction of the eutectic point and the calculation of the ideal eutectic point.

Density of CA:Gly

The densities of 1:2 and 1:4 CA:Gly at 20 °C were measured using an Anton Paar DMA 4500 M.

Table S7: Densities of 1:2 and 1:4 CA:Gly at 20 °C.

Solvent	Density / g/cm^3
1:2 CA:Gly	1.3932
1:4 CA:Gly	1.3491

References

1. SASview software. www.sasview.org (accessed 08.04.2024).
2. Guinier, A.; Fournet, G., *Small-Angle Scattering of X-Rays*. 1st ed. ed.; Wiley: New York, 1955.
3. Kotlarchyk, M.; Chen, S. H., Analysis of small angle neutron scattering spectra from polydisperse interacting colloids. *J Chem. Phys.* **1983,** *79* (5), 2461-2469.
4. Sanchez-Fernandez, A.; Arnold, T.; Jackson, A. J.; Fussell, S. L.; Heenan, R. K.; Campbell, R. A.; Edler, K. J., Micellization of alkyltrimethylammonium bromide surfactants in choline chloride: glycerol deep eutectic solvent. *Phys. Chem. Chem. Phys.* **2016,** *18* (48), 33240-33249.

5. Sanchez-Fernandez, A.; Edler, K. J.; Arnold, T.; Heenan, R. K.; Porcar, L.; Terrill, N. J.; Terry, A. E.; Jackson, A. J., Micelle structure in a deep eutectic solvent: a small-angle scattering study. *Phys. Chem. Chem. Phys.* **2016,** *18* (20), 14063-14073.

6. Glatter, O.; Fritz, G.; Lindner, H.; Brunner-Popela, J.; Mittelbach, R.; Strey, R.; Egelhaaf, S. U., Nonionic Micelles near the Critical Point: Micellar Growth and Attractive Interaction. *Langmuir* **2000,** *16* (23), 8692-8701.

7. Jacques, D. A.; Trewhella, J., Small-angle scattering for structural biology-Expanding the frontier while avoiding the pitfalls. *Protein Sci.* **2010,** *19* (4), 642-657.

8. Manasi, I.; Andalibi, M. R.; Atri, R. S.; Hooton, J.; King, S. M.; Edler, K. J., Self-assembly of ionic and non-ionic surfactants in type IV cerium nitrate and urea based deep eutectic solvent. *J. Chem. Phys.* **2021,** *155* (8), 084902.

9. Dolan, A.; Atkin, R.; Warr, G. G., The origin of surfactant amphiphilicity and self-assembly in protic ionic liquids. *Chem. Sci.* **2015,** *6* (11), 6189-6198.

10. Sanchez-Fernandez, A.; Hammond, O. S.; Jackson, A. J.; Arnold, T.; Doutch, J.; Edler, K. J., Surfactant-Solvent Interaction Effects on the Micellization of Cationic Surfactants in a Carboxylic Acid-Based Deep Eutectic Solvent. *Langmuir* **2017,** *33* (50), 14304-14314.

11. López-Barrón, C. R.; Wagner, N. J., Structural Transitions of CTAB Micelles in a Protic Ionic Liquid. *Langmuir* **2012,** *28* (35), 12722-12730.

12. Gerlach, T.; Müller, S.; de Castilla, A. G.; Smirnova, I., An open source COSMO-RS implementation and parameterization supporting the efficient implementation of multiple segment descriptors. *Fluid Phase Equilibria* **2022,** *560*, 113472.

13. "Condensed Phase Heat Capacity Data" by Eugene S. Domalski and Elizabeth D. Hearing and "Thermochemical Data" by Donald R. Burgess, Jr. In *NIST Chemistry WebBook, NIST Standard Reference Database Number 69*, Linstrom, P. J.; Mallard, W. G., Eds. National Institute of Standards and Technology: Gaithersburg MD, 20899.

14. Neese, F., Software update: The ORCA program system—Version 5.0. *WIREs Computational Molecular Science* **2022,** *12* (5), e1606.

15. Müller, S.; Nevolianis, T.; Garcia-Ratés, M.; Riplinger, C.; Leonhard, K.; Smirnova, I., Predicting solvation free energies for neutral molecules in any solvent with openCOSMO-RS. *Fluid Phase Equilibria* **2025,** *589*, 114250.
